# Regulation of neovasculogenesis in co-cultures of aortic adventitial fibroblasts and microvascular endothelial cells by cell-cell interactions and TGF-β/ALK5 signaling

**DOI:** 10.1371/journal.pone.0244243

**Published:** 2020-12-28

**Authors:** Rebecca A. Scott, Eric W. Fowler, Xinqiao Jia, Kristi L. Kiick, Robert E. Akins

**Affiliations:** 1 Department of Materials Science and Engineering, University of Delaware, Newark, Delaware, United States of America; 2 Nemours—Alfred I. duPont Hospital for Children, Wilmington, Delaware, United States of America; 3 Delaware Biotechnology Institute, University of Delaware, Newark, Delaware, United States of America; Hungarian Academy of Sciences, HUNGARY

## Abstract

Adventitial fibroblasts (AFs) are critical mediators of vascular remodeling. However, the contributions of AFs towards development of vasculature and the specific mechanisms by which these cells regulate physiological expansion of the vasa vasorum, the specialized microvasculature that supplies nutrients to the vascular wall, are not well understood. To determine the regulatory role of AFs in microvascular endothelial cell (MVEC) neovasculogenesis and to investigate the regulatory pathways utilized for communication between the two cell types, AFs and MVECs were cultured together in poly(ethylene glycol)-based hydrogels. Following preliminary evaluation of a set of cell adhesion peptides (AG10, AG73, A2G78, YIGSR, RGD), 7.5wt% hydrogels containing 3 mM RGD were selected as these substrates did not initiate primitive tubule structures in 3D MVEC monocultures, thus providing a passive platform to study AF-MVEC interaction. The addition of AFs to hydrogels promoted MVEC viability; however, increasing AF density within hydrogels stimulated MVEC proliferation, increased microvessel density and size, and enhanced deposition of basement membrane proteins, collagen IV and laminin. Importantly, AF-MVEC communication through the transforming growth factor beta (TGF-β)/activin receptor-like kinase 5 (ALK5) signaling pathway was observed to mediate microvessel formation, as inhibition of ALK5 significantly decreased MVEC proliferation, microvessel formation, mural cell recruitment, and basement membrane production. These data indicate that AFs regulate MVEC neovasculogenesis and suggest that therapeutics targeting the TGF-β/ALK5 pathway may be useful for regulation of vasculogenic and anti-vasculogenic responses.

## Introduction

The vasa vasorum is a specialized microvasculature that supplies the outer layers of large blood vessels with oxygen and nutrients [1]. Developmentally, this microvasculature, which comprises networks of lumenized endothelial cell (EC) conduits surrounded by connective tissue and pericytes or other mural cells, is established early in gestation [2]. The vasa vasorum is modified and expanded with normal development [3], and significant alterations, especially in the adventitial vasa vasorum, have been observed in several vascular diseases. Atherosclerosis [4], arterial stenosis [5], and pulmonary arterial hypertension [6] all exhibit hallmarks of increased adventitial vasa vasorum density, which is thought to facilitate the inflammatory cell trafficking associated with disease progression [7]. Interestingly, the vasa vasorum also plays a critical role in the success of revascularization procedures [8]. For example, damage to the adventitial microvasculature of saphenous vein grafts during coronary artery bypass procedures can result in neointimal hyperplasia and atheroma formation [9]. The vasa vasorum clearly plays important roles in physiologic and pathophysiologic processes in blood vessels [10], and there is a need to better understand the cellular mechanisms involved in the formation of functional microvessels for the development of interventions that support healthy vasa vasorum.

The importance of ECs in the generation of new microvasculature has been a primary focus for numerous researchers. ECs create new vasculature in response to hypoxia, which arises when the microvasculature is severed or as the size or diffusion parameters of tissue are altered [11]. In addition, communication between ECs and non-ECs plays a critical role in directing neovascularization [12]. For example, secretion of pro-angiogenic growth factors by non-ECs, including smooth muscle cells (SMCs), macrophages, and mesenchymal stem cells (MSCs), promotes *de novo* formation of microvessels [13–15]. Similarly, adventitial fibroblasts (AFs), a principal cell type found within vascular adventitia, have been shown to play an important role in regulating development and expansion of the vasa vasorum [16]. Following vascular injury, AFs become activated, often differentiating into myofibroblasts, and secrete growth factors that contribute to vascular remodeling [17]. The phenotypic switch of AFs to myofibroblasts has been found to correspond to an increased number of vasa vasorum, with AFs found proximal to microvessels during vascular expansion [18]. Li et. al. recently demonstrated that secretion of the pro-angiogenic growth factor, vascular endothelial growth factor (VEGF-A), by activated AFs resulted in vasa vasorum-associated neointimal hyperplasia [19]. Although upregulation of select growth factors has been suggested [20], the precise mechanism by which AFs regulate the number of microvessels and instigate *de novo* formation of vasa vasorum remains largely unknown.

Transforming growth factor beta 1 (TGF-β1), which is produced by both AFs and ECs, plays a critical role during vascular development and remodeling [21, 22]. This multifunctional cytokine differentially regulates both activation and differentiation of ECs via two TGF‐β type I receptors, activin receptor-like kinase 1 (ALK1) and ALK5 [23]. Though both ALK1 and ALK5 are activated upon TGF-β1 simulation, recent observations suggest that ALK1 pathway activation, through SMAD-1/5 phosphorylation, initially turns on migratory and proliferative target genes in ECs, while simultaneously limiting signaling through ALK5, culminating in increased EC proliferation, migration, and tube formation [23]. However, following prolonged TGF-β stimulation, ALK1 signaling diminishes and increased activation of the ALK5 pathway is observed via SMAD-2/3 phosphorylation, resulting in inhibition of EC proliferation, migration, and vessel maturation [23]. While ALK1 expression is primarily restricted to ECs, ALK5 expression is fairly ubiquitous across cell types [24]. As such, TGF-β signaling through ALK5 in mural cells leads to the recruitment, extracellular matrix (ECM) production, and differentiation of these cells, culminating in vessel stabilization [25, 26]. However, the specific contributions of TGF-β/ALK5 signaling towards AF-mediated *de novo* vasa vasorum formation remain unknown.

*In vitro* cell culture systems comprising synthetic biomaterials provide valuable platforms for studying cell interactions and signaling cascades. Such platforms could be of great use for understanding signaling mechanisms involved in AF regulation of microvessel formation, in providing insight into signaling mechanisms regulating neovasculogenesis, and in identifying potential therapeutic approaches for regulating maladaptive vascular remodeling. Biomaterials offer significant versatility with respect to tuning of mechanical stiffness, presentation of specific cell-binding and degradation domains, and incorporation of cytokines to direct cell behavior [27]. In particular, poly(ethylene glycol) (PEG)-based hydrogels are biologically inert and serve as a highly tailorable blank canvas while also offering initial autonomous control over mechanical and biochemical properties [28]. Importantly, PEG-based substrates have been fabricated to comprise cues important for supporting vasculogenesis and angiogenesis [29, 30], to study cell-cell and cell-ECM interactions [31, 32], and to investigate important signaling pathways involved in vascular cell function [33, 34].

In this study, we utilized PEG-based hydrogels as a platform to investigate the influence of aortic adventitial fibroblasts (AoAFs) on microvascular endothelial cell (MVEC) vasculogenesis. Following evaluation of the cell adhesion peptides (AG10, AG73, A2G78, YIGSR, RGD), hydrogels containing 3 mM RGD were selected as these substrates did not initiate primitive tubule structures in MVEC monocultures, thus providing a passive platform to study AoAF-MVEC interaction. Human MVECs and AoAFs were encapsulated in PEG-based hydrogels and the impact of cell ratio and alterations in physicochemical properties of the hydrogels were analyzed. Furthermore, microvessel formation was investigated by quantifying cell viability, proliferation, and basal, as well as neovessel, characteristics. Previous work has indicated the importance of TGF-β1 signaling on microvessel formation and vessel wall integrity [35]. Here, we assessed the role of ALK5, which is ubiquitously expressed among cells, in mediating AoAF-MVEC interactions. The present study was conducted to better elucidate the role of AoAFs in directing MVEC neovasculogenesis to support the development of therapies to improve vasa vasorum health in revascularization and cardiovascular disease.

## Materials and methods

### Cell maintenance

De-identified human cardiac microvascular endothelial cells (MVEC) and aortic adventitial fibroblasts (AoAF) were obtained from a commercial source with Institutional Review Board approval. MVECs (35 yo male donor; Lonza, Walkersville, MD) were cultured in microvascular endothelial cell growth medium-2 (EGM2-MV), which comprised microvascular endothelial cell basal medium supplemented with 5% fetal bovine serum (FBS), and a proprietary cocktail of hydrocortisone, basic fibroblast growth factor (bFGF), vascular endothelial cell growth factor, insulin-like growth factor 1, ascorbic acid, and epidermal growth factor (all from Lonza), and used between passage numbers 6 and 8 for all assays. AoAF (de-identified 53 yo male donor; Lonza) were cultured in fibroblast growth medium (FGM), which comprised fibroblast basal medium supplemented with 5% FBS, bFGF, and insulin (all from Lonza), and used between passage numbers 6 and 8 for all assays. Cells were maintained at 37°C with 5% CO_2_.

The presence of a consistent cell phenotype in MVEC cultures was validated by examining cell morphology, proliferative capacity, and verifying, via immunofluorescent staining, that greater than 90% of the cells were positive for CD31, a marker of endothelial cells, at each passage. AoAF cultures were validated by examining cell morphology, proliferative capacity, and verifying, via immunofluorescent staining, that fewer than 10% of the cells were positive for α-smooth muscle actin (αSMA), a marker of myofibroblast transdifferentiation, at each passage.

### Peptide synthesis & characterization

The bis-maleimide (MI), end-functionalized, enzymatically-degradable crosslinker peptide (MI-GG**PQ**GIRGQGK(MI)G; PQ-MI_2_) and monomaleimide-functionalized RGD peptide (MI-KGG**RGD**SPG; RGD-MI) were synthesized using a Focus Xi automated peptide synthesizer (AAPPTec, Louisville, KY) with standard Fmoc chemistry, at 0.5 mmol scale using Rink-Amide resin (ChemPep Inc, Wellington, FL), as in prior studies [34, 36]. PQ-MI_2_ and RGD-MI were synthesized with Fmoc-Lys(Dde)-OH (ChemPep Inc). Following solid phase synthesis, the N-terminal amine on the RGD-MI and PQ-MI_2_ peptides were deprotected with a 20% (v/v) piperidine (Sigma Aldrich, St. Louis, MO)/N,N-dimethylformamide (DMF; Fisher Scientific, Waltham, MA) solution. The N-terminal amine on RGD-MI was then acetylated via acetic anhydride (25 mmol; Sigma Aldrich)/N,N-diisopropylethylamine (DIPEA; 25 mmol; Sigma Aldrich) in DMF. Deprotection of lysine ε-amine was then performed for both peptides with hydroxylamine hydrochloride (18 mmol; Sigma Aldrich) and imidazole (13.5 mmol; Sigma Aldrich) in 5:1 N-methyl-2-pyrrolidone (NMP; Fisher Scientific):dichloromethane (DCM; Fisher Scientific). Following, the N-terminal glycine and lysine ε-amines were coupled with 3-maleimidopropionic acid (4 mmol; Sigma Aldrich) using 1-[bis(dimethylamino)methylene]-1H-1,2,3-triazolo[4,5-b]pyridinium 3-oxide hexafluorophosphate (4 mmol; AAPPTec) and DIPEA (8 mmol) in DMF.

Laminin-derived peptides were prepared using a Liberty Blue automated peptide synthesizer (CEM, Mathews, NC) with microwave assisted Fmoc mediated solid phase synthesis, at 0.25 mmol scale using Rink-Amide ProTide resin (CEM). AG10 (MI-KDRSG**NRWHSIYITRFG**; AG10-MI), AG73 (MI-KDRSG**RKRLQVQLSIRT**; AG73-MI), and A2G78 (MI-KDRSG**GLLFYMARINHA**; A2G78-MI) peptides were synthesized with Boc-Lys(Fmoc)-OH (AAPPTec) at the N-terminus and the ε-amine was used to incorporate maleimide functionality. Deprotection of the N-terminal lysine was performed with a 20% (v/v) piperidine/DMF solution. Following, lysine ε-amines were coupled with {2-[2-(2,5-dioxo-2,5-dihydro-1H-pyrrol-1-yl)ethoxy]ethoxy}acetic acid (Chem-Impex, Wood Dale, IL) using N,N,N′,N′-tetramethyl-O-(1H-benzotriazol-1-yl)uronium hexafluorophosphate (HBTU; 1.0 mmol; ChemPep)/DIPEA (2.0 mmol) in DMF. The YIGSR peptide (MI-GGG**YIGSR**; YIGSR-MI) was maleimide functionalized by reacting the N-terminal glycine amine with 4-malimeidobutyric acid (1.0 mmol; TCI Chemicals, Portland, OR) using HBTU (1.0 mmol)/DIPEA (2.0 mmol) in DMF.

All maleimide-functionalized peptides were cleaved from resin using a solution of trifluoroacetic acid (TFA; Sigma Aldrich)/triisopropylsilane (TIPS; Sigma Aldrich)/H_2_O (95%:2.5%:2.5%, v/v/v), precipitated in cold diethyl ether, and dried *in vacuo* prior to purification by reverse-phase high-performance liquid chromatography (HPLC, Waters, Millford, MA), using a BEH130 Preparative C18 10-μm column (XBridge, Waters). Crude peptides were dissolved in water containing 0.1% (v/v) TFA and filtered (0.2-μm filter, Corning) in preparation for HPLC injection. PQ-MI_2_, RGD-MI, and YIGSR-MI were subjected to an elution gradient of 100% (v/v) solvent A (0.1% (v/v) TFA in Milli-Q water) to 70% (v/v) solvent A within 30 min; solvent B consisted of acetonitrile with 0.1% (v/v) TFA. AG10-MI and AG73-MI were purified similarly, but with an adjustment of gradient to 50% solvent A. Ultraviolet-visible detection at 214 nm (Waters 2489, Waters) was used to monitor and collect eluted peptide fractions. Target product M/Z was verified using a Waters Xevo G2-XS QTof mass spectrometer. PQ-MI_2_ calculated exact mass = 1412.5 Da, observed [M+H]^+^ = 1413.7; RGD-MI calculated exact mass = 1022.1 Da, observed [M+H]^+^ = 1022.4; AG10-MI calculated exact mass = 2317.4 Da, observed [M+2H]^+2^ = 1159.6, [M+3H]^3+^ = 773.4, [M+4H]^4+^ = 580.3, [M+5H]^5+^ = 464.4; AG73-MI calculated exact mass = 2265.5 Da, observed [M+2H]^+2^ = 1133.7, [M+3H]^3+^ = 756.1, [M+4H]^4+^ = 567.3, [M+5H]^5+^ = 454.1; A2G78-MI calculated exact mass = 2173.3 Da, observed m/z [M+2H]^+2^ = 1087.5, [M+3H]^3+^ = 725.4, [M+4H]^4+^ = 544.3, [M+5H]^5+^ = 434.1; YIGSR-MI calculated exact mass = 929.4 Da, observed [M+H]^+^ = 930.7. Peptides were stored, under argon, as a lyophilized powder at −20°C until use.

### Hydrogel formation

Degradable hydrogels were formed by crosslinking four-arm thiol-functionalized poly(ethylene glycol) (PEG-SH_4_; f = 4, Mn 10,000 g/mol, JenKem Technology, Allen, TX) with PQ-MI_2_ and mono-maleimide pendent cell adhesion peptides. Hydrogels were prepared at concentrations of 7.5wt% and contained either 3 mM RGD-MI, 3 mM YIGSR-MI, or 3 mM of laminin peptide cocktail (0.1 mM AG10-MI, 0.5 mM AG73-MI, 0.4 mM RGD-MI, and 2 mM YIGSR-MI). Substrates were crosslinked at a thiol to maleimide stoichiometric ratio of 1:1 and 7.5wt% hydrogels comprised 5.8 mM PEG-SH_4_ and 10.1 mM PQ-MI_2_. To form hydrogels, PEG-SH_4_, PQ-MI_2_, and the mono-maleimide pendent cell adhesion peptides were independently dissolved in buffer (10 mM sodium phosphate monobasic monohydrate, 5 mM citric acid trisodium salt (anhydrous), 140 mM sodium chloride, pH 4.8) and sterilized by passage through a 0.2 μm nonpyrogenic PVDF filter (Sigma Aldrich). Maleimide- and thiol-containing solutions, along with 10 mM 4-(2-hydroxyethyl)-1-piperazineethanesulfonic acid (HEPES; Gibco, Gaithersburg, MD) in Hank’s Balanced Salt Solution (HBSS; 1/5 of the final hydrogel volume; pH 7.4; Gibco), were mixed together via pipetting.

Non-degradable hydrogels were formed by crosslinking PEG-SH_4_ with four-arm maleimide-functionalized PEG (PEG-MI_4_; f = 4, Mn 10,000 g/mol, JenKem Technology) and RGD-MI. Hydrogels were prepared at a concentration of 10wt% and crosslinked at a thiol to maleimide stoichiometric ratio of 1:1, such that 10wt% hydrogels comprised 5 mM PEG-SH_4_, 2 mM PEG-MI_4_, and 3 mM RGD-MI. To form hydrogels, PEG-SH_4_, PEG-MI_4_, and RGD-MI were independently dissolved in buffer (10 mM citrate buffer, pH 4.5) and sterilized by passage through a 0.2 μm nonpyrogenic PVDF filter. Maleimide- and thiol-containing solutions were then mixed together with 10 mM HEPES in HBSS (1/5 of the final hydrogel volume) via pipetting.

### Rheological characterization of hydrogels

The mechanical properties of the hydrogels were characterized via bulk oscillatory rheology (AR-G2, TA instruments). Briefly, the hydrogel precursor solutions were thoroughly mixed and added via pipette to a cylindrical mold (30 μL; diameter = 4.6 mm, thickness = 1.8 mm) and allowed to crosslink for 20 mins at room temperature. Hydrogels were gently removed from the mold and subsequently immersed in co-culture medium, comprising 1:1 EGM2-MV:FGM (1.5 mL), and incubated at 37°C with 5% CO_2_ overnight. The hydrogels were then transferred to a 20-mm diameter stainless steel, parallel plate geometry after 24 hours, and the equilibrium shear storage moduli of the swollen hydrogels were measured. Time sweep measurements were obtained within the linear viscoelastic regime using 2% constant strain and 2 rad/s angular frequency. A normal force of 0.2 N was applied to prevent slip during measurement.

### Encapsulation of vascular cells in hydrogels

To encapsulate cells in hydrogels, MVECs and AoAFs were suspended in 10 mM HEPES in HBSS and mixed via gentle pipetting into the solution of hydrogel precursor polymers prior to crosslinking. For MVEC monocultures, MVECs were encapsulated to achieve 3x10^6^ cells/mL hydrogel. For AoAF monocultures, AoAFs were encapsulated to achieve 1x10^6^ or 3x10^6^ cells/mL hydrogel. For MVEC and AoAF co-cultures, MVECs were encapsulated to achieve 3x10^6^ MVECs/mL hydrogel and AoAFs were encapsulated to achieve either 1x10^6^ or 3x10^6^ AoAFs/mL hydrogel, as indicated in the text. Once mixed via gentle pipetting, 10 μL volumes of degradable hydrogel were added to the surface of an 8 well Nunc^TM^ Lab-Tek^TM^ chambered coverglass and allowed to crosslink for 20 mins at room temperature. As pilot assessments in 2D showed no differences in adhesion or proliferation for AoAF or MVEC monocultures grown in each cell type’s specific medium or co-culture medium (**S1 Fig**), hydrogels were then immersed in co-culture medium (400 μL), comprising 1:1 EGM2-MV:FGM, and incubated at 37°C with 5% CO_2_. In studies where TGF-β superfamily pathways were investigated, hydrogels were immersed in co-culture medium supplemented with A83-01 (1 μM; Cayman Chemical, Ann Arbor, MI). A83-01 serves as a potent inhibitor of ALK5, as well as ALK4 and ALK7, by blocking the phosphorylation of SMAD2/3 [37]. Medium was replaced every 3 days over the duration of these experiments, with conditioned medium collected and stored at -80°C for further analysis. In experiments testing the impact of AoAF-generated diffusible factors on MVEC viability, MVEC monocultures were alternatively immersed in AoAF-conditioned medium (400 μL), which was collected from AoAF monocultures (3x10^6^ cells/mL hydrogel) every 3 days over the first 9 days of culture.

In studies where layered hydrogels were utilized, a 5 μL volume of degradable hydrogel, containing 3x10^6^ MVECs/mL of hydrogel, was prepared as described above, deposited on the surface of an 8 well Nunc^TM^ Lab-Tek^TM^ chambered coverglass, and allowed to crosslink for 10 mins at room temperature. Next, a 5 μL volume of either non-degradable hydrogel or degradable hydrogel, containing 3x10^6^ AoAFs/mL of hydrogel, was prepared as described above and deposited on top of the first MVEC-laden hydrogel layer. The layered hydrogel was allowed to crosslink for 15 additional minutes at room temperature, and then was subsequently immersed in co-culture medium (400 μL) and incubated at 37°C with 5% CO_2_. Medium was replaced every 3 days over the duration of these experiments.

### Cell viability

The viability of MVECs and AoAFs in hydrogels was assessed using the LIVE/DEAD™ viability/cytotoxicity kit (ThermoFisher Scientific, Waltham, MA) on days 1 and 7. Briefly, acetomethoxy-calcein (calcein-AM; 2 μM) and ethidium homodimer-1 (2 μM) in phosphate-buffered saline (PBS, pH 7.4) were added to cell-laden hydrogels; the hydrogels were incubated at 37°C for 15 mins and washed in PBS. Cells were visualized using Zeiss 710 confocal microscope (Carl Zeiss, Thornwood, NY) with a 10x EC Plan-Neofluar 0.3 N.A. objective to assess the presence of calcein-AM, which fluoresces green to indicate live cells capable of enzymatic de-esterification of calcein-AM, and the presence of nucleic acid intercalated ethidium, which fluoresces red to indicate dead cells unable to exclude the dye from the cell nucleus. Three scan volumes were completed in distinct locations for each culture with an xy area of 0.72 mm^2^ and a z depth of 100 μm (4.65 μm per step).

### Immunostaining

For assessment in 3D cultures, PEG hydrogels were removed from culture medium and fixed with 4% (v/v) paraformaldehyde (Sigma Aldrich) in PBS (pH 7.4) for 1 hr, permeabilized with 0.2% (v/v) Triton X-100 (Sigma Aldrich) for 45 min, and blocked with 3% (w/v) bovine serum albumin (Sigma Aldrich) in PBS for 45 min. Antibodies are listed in **S1 Table**. Samples were stained with primary antibodies for 4 hrs at room temperature followed by 68 hrs at 4°C with shaking, washed extensively with PBS containing 0.05% (v/v) Tween-20 (Sigma Aldrich), and incubated with secondary antibodies, Alexa Fluor^TM^ 568 Phalloidin (1:100; Life Technologies, Carlsbad, CA), and the nuclear stain Hoechst 33258 (1:1000; Life Technologies) for 2 hrs at room temperature, followed by 46 hrs at 4°C, with shaking. Cells were visualized using a Zeiss 710 confocal microscope with either a 20x Plan-Apochromat 0.8 N.A. or a 40x C-Apochromat 1.2 N.A. objective. Three scan volumes were completed in distinct locations for each culture. For 20x objective, an xy area of 0.18 mm^2^ and a z depth of 100 μm (1.44 μm per step) was used; for the 40x objective, an xy area of 0.05 mm^2^ and a z depth of 20 μm (0.42 μm per step).

### Proliferation & measurement of multi-cellular network characteristics

On days 1, 7, 14, and 28, cells in hydrogels were fixed, stained for CD31, F-actin, and nuclei, and visualized via confocal microscopy, as described above. Proliferation of MVECs and AoAFs were evaluated in mono- and co-cultures by enumerating cell nuclei in each of the z-stacks collected within hydrogel cultures and converting to cells/mm^3^. To distinguish between MVEC and AoAF cell types in co-cultures, CD31-positive cells were considered to be MVECs, while CD31-negative cells staining positively for F-actin only were identified as AoAFs. ImageJ (http://imagej.nih.gov/ij/; provided in the public domain by the National Institutes of Health, Bethesda, MD, USA) was used for image assessment and quantification.

To assess MVEC network formation, a maximum intensity projection (MIP) of CD31 fluorescence was created using Zen Black (Carl Zeiss) for each z-stack acquired. MIPs were then used to estimate network parameters including the number of junctions, number of MVEC tubules, total tubule length (sum of the lengths of all segments), and tubule diameter using the Angiogenesis Analyzer plug-in on ImageJ [38]. Lumen formation in the 3D co-culture systems was assessed by reconstructing merged images of CD31 and Hoechst 33258 into a 3D configuration. Slice views of the MVEC structures from both x-z axis and y-z axis were then used to analyze the formation of luminal structures.

### Statistics

Data are expressed as the mean ± standard error of the mean, unless otherwise noted. Details regarding the specific test utilized to evaluate each experiment are indicated in figure captions. In brief, assumptions of residual normality (Shapiro–Wilks test) and homoscedasticity (Bartlett's test) were first verified for each group. Following confirmation that assumptions for parametric analysis were met, statistical significance was analyzed by performing either a Student's *t*‐test, one‐way ANOVA, or two‐way ANOVA, as appropriate, where *p* < 0.05 was considered significant. In cases where a one‐way or two‐way ANOVA was used, if the F‐test revealed significant statistical differences at the 0.05 level, pairwise comparisons were made using Tukey HSD post‐hoc. Statistical interpretations were made using JMP Pro 14 (SAS Institute Inc.).

## Results

### Development of passive 3D PEG hydrogels

To establish a 3D PEG-based hydrogel system that could serve as a passive platform upon which the contributions of AoAFs towards regulating MVEC microvessel formation could be investigated, cell adhesive peptide sequences that support MVEC-matrix interactions were first identified. Multiple biologically active laminin peptides, including AG73, AG10, A2G78, and YIGSR, which target multiple ECM receptors, such as α6β1, α3β1, α-dystroglycan, 67-kDa laminin receptor, and syndecan [39–41], and the fibronectin-derived peptide, RGD, which target α5β1 and αvβ3 [42], interact with microvascular cells. As such, these peptide motifs were evaluated in a subset of preliminary experiments that investigated the potential of these ligands to support MVEC-matrix interactions. Peptide competition assays revealed that addition 0.2 mM of AG10, AG73, or YIGSR peptides, but not A2G78, to MVECs on Matrigel disrupted tube formation in an *in vitro* angiogenesis assay (**S2 Fig**) [43]. In a separate set of preliminary experiments, incorporation of RGD within non-degradable PEG-based hydrogels was observed to promote MVEC adhesion on 2D substrates (**S3 Fig**). Based on these preliminary results, the laminin-derived peptides, AG10, AG73, and YIGSR, as well as the fibronectin-derived RGD peptide, were selected for further evaluation in PEG-based hydrogels.

**Fig 1:**
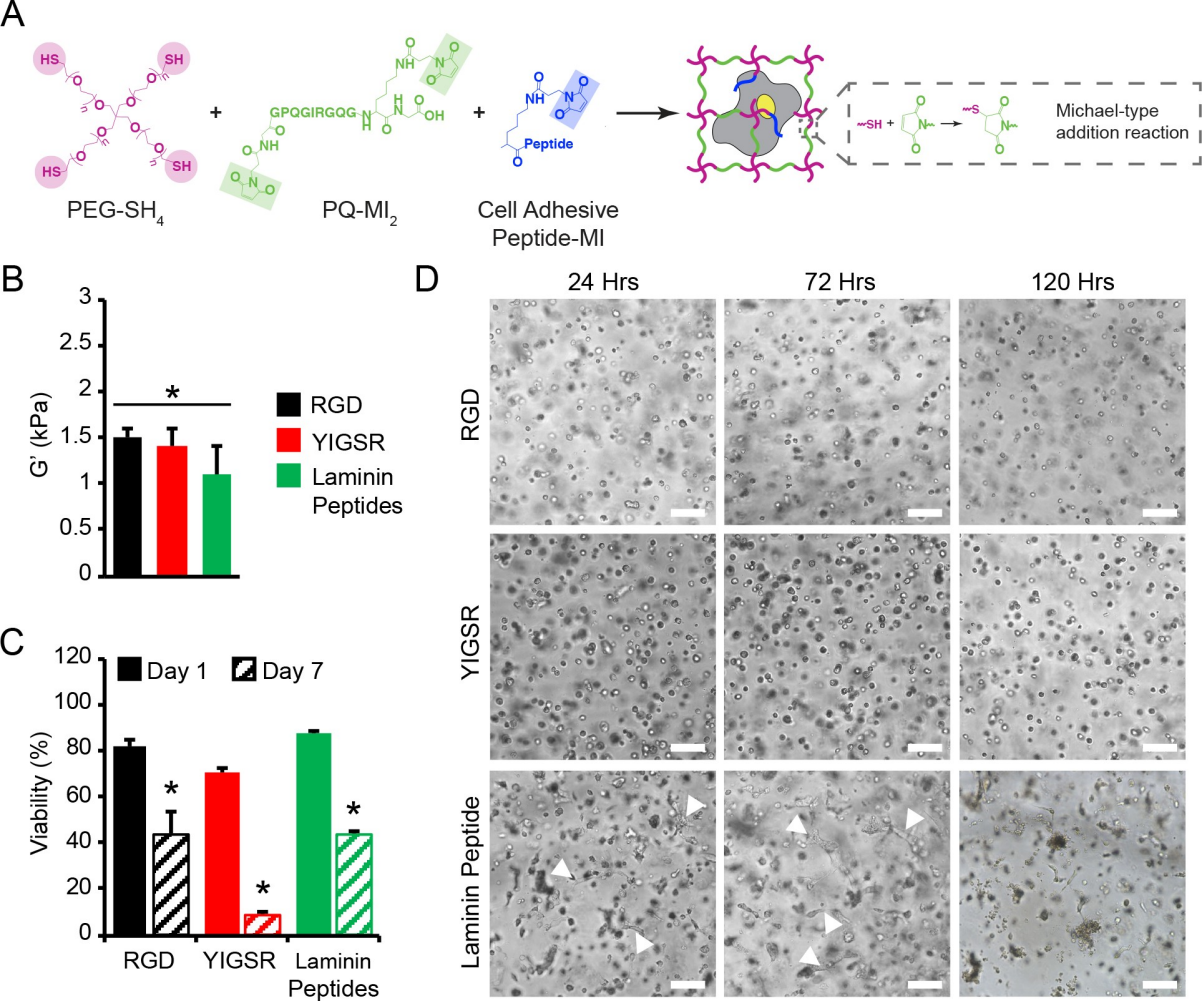
Characterization of PEG-based hydrogels with cell adhesion peptides and impact of hydrogels on MVEC spreading and viability. (**A**) Reaction scheme for enzymatically degradable hydrogel formation with cell adhesion peptides. (**B**) The initial equilibrium storage moduli (G’, kPa), evaluated by oscillatory shear rheology after 24 h, was similar across hydrogels containing 3 mM RGD, 3 mM YIGSR, or 3 mM laminin peptide cocktail. (**C**) Over 70% viability was observed for vascular cells encapsulated PEG hydrogels after 1 day of encapsulation. Viability in MVEC monocultures significantly decreased after 7 days of culture. (**D**) MVEC monocultures did not form tubule-like structures in 7.5wt% hydrogels containing 3 mM RGD or 3 mM YIGSR within 120 hrs post-encapsulation in hydrogels. However, in 7.5wt% hydrogels containing 3 mM laminin-derived peptides, monocultures of MVECs initially formed tubule-like structures within 72 hrs; however, structures digressed by 120 hrs post-encapsulation in hydrogels. White arrows point to tubule-like structures. Scale bar = 100 μm. In **B-C**: data are represented as the mean ± SEM, with *n* = 3 biological replicates per condition. In B: a one-way ANOVA was used to detect statistical significance, ns = no significance. **C**: a two-way ANOVA, followed by a Tukey HSD post hoc test, was used to detect statistical significance, *p<0.05 for day 7 relative to day 1, within an individual hydrogel system.

To examine the role of select laminin- and fibronectin- derived peptides on MVEC behavior, hydrogels were formed via a biocompatible Michael-type addition reaction, under physiologic pH and salt concentrations, using thiol-functionalized PEG macromers (PEG-SH_4_) and bis-maleimide, end-functionalized MMP-sensitive crosslinker peptides (PQ-MI_2_) to obtain highly elastic hydrogels that accommodate cell-mediated matrix remodeling (**Fig 1A**). In addition, either 3 mM RGD-MI, 3 mM YIGSR-MI, or 3 mM of a laminin peptide cocktail (2 mM YIGSR-MI, 0.5 mM AG73-MI, 0.4 mM RGD-MI, 0.1 mM AG10-MI) was incorporated into the polymer matrix (**S2 Table**). Oscillatory shear rheology was utilized to measure the viscoelastic properties of the hydrogel formulation via dynamic time sweep assays in the linear viscoelastic regime. The equilibrium shear storage modulus for acellular 7.5wt% containing 3 mM RGD-MI, 3 mM YIGSR-MI, or 3 mM of laminin peptide cocktail into the polymer matrix was 1.5 ± 0.1 kPa, 1.4 ± 0.2 kPa, and 1.1 ± 0.3 kPa, respectively, after 24 h of incubation at 37°C in co-culture medium (**Fig 1B**).

MVECs were encapsulated in degradable PEG-based hydrogels at a density of 3x10^6^ cells/mL hydrogel, similar to published studies evaluating MVECs in 3D [44]. Cell were homogeneously distributed throughout the hydrogels and exhibited high viability after 24 hrs (RGD-MI: 82.3% ± 2.6%; YIGSR-MI: 70.2% ± 1.9%; laminin peptide cocktail: 87.9% ± 0.5%; **Fig 1C and S4 Fig**). Minimal spreading was observed for MVECs encapsulated in PEG hydrogels containing either RGD or YIGSR over the 5 days of culture (**Fig 1D**). Conversely, PEG hydrogels containing the laminin peptide cocktail supported formation of primitive tubule structures by MVECs, which were apparent after 3 days of culture; however, tubules were unstable and dissipated within 5 days. Regression of the MVEC structures corresponded to decreased viability in laminin peptide hydrogels, where 32.1% ± 0.9% of cells were viable after 7 days of culture. Decreased cell viability was also observed in hydrogels containing RGD or YIGSR after 7 days of culture (RGD-MI: 43.7% ± 9.5%; YIGSR-MI: 8.9% ± 0.7%). Ultimately, as PEG-based hydrogels containing RGD did not initiate primitive MVEC tubule structures, and sustained greater viability compared to YIGSR-laden hydrogels, this model system was selected to evaluate the role of AoAFs in directing MVEC network formation.

### AoAFs enhance MVEC viability in 3D PEG hydrogels

**Fig 2:**
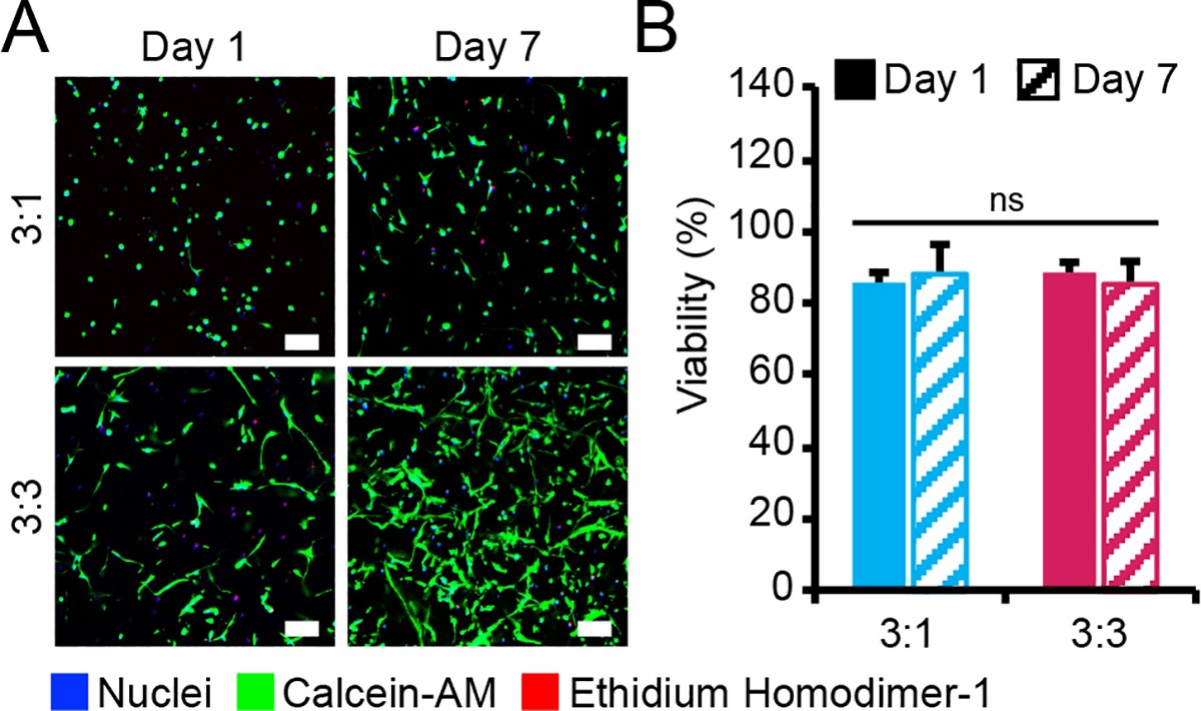
Viability of MVEC:AoAF co-cultures in degradable PEG-based hydrogels. (**A**) Representative live/dead images of 3:1 and 3:3 MVEC:AoAF co-cultures in degradable 7.5wt% hydrogels with 3 mM RGD after 1 and 7 days of culture. Green indicates live cell bodies and red indicates nuclei in necrotic cells. Scale bar = 100 μm. (**B**) Over 82% viability was observed for vascular cells encapsulated PEG hydrogels after 1 day of encapsulation and viability was maintained (>85%) in 3:1 and 3:3 co-cultures. In **B**: data are represented as the mean ± SEM, with *n* = 3 biological replicates per condition. A two-way ANOVA, followed by a Tukey HSD post hoc test, was used to detect statistical significance, ns = no significance.

To determine whether the addition of AoAFs to 3D PEG hydrogels facilitated MVEC networking spreading, different ratios of the two cell types were encapsulated in degradable 7.5wt% hydrogels containing 3 mM RGD-MI. Co-cultures were initiated at either a 3:1 (i.e., 3x10^6^ MVECs/mL hydrogel, 1x10^6^ AoAFs/mL hydrogel) or a 3:3 (i.e., 3x10^6^ MVECs/mL hydrogel, 3x10^6^ AoAFs/mL hydrogel) MVEC:AoAF ratio. High cell viability was observed (3:1: 86.0% ± 3.3%; 3:3: 88.5% ± 1.7%) after 24 hrs in both co-culture systems, with vascular cells homogeneously distributed throughout the hydrogel (**Fig 2**). After 7 days of culture, viability in the 3:1 and 3:3 co-cultures remained high (3:1: 88.1% ± 6.6%; 3:3: 85.0% ± 4.7%), with vascular cell spreading and the appearance of cellular networks observed in both co-culture systems after 7 days of culture.

### AoAFs promote microvessel formation in PEG hydrogels

**Fig 3:**
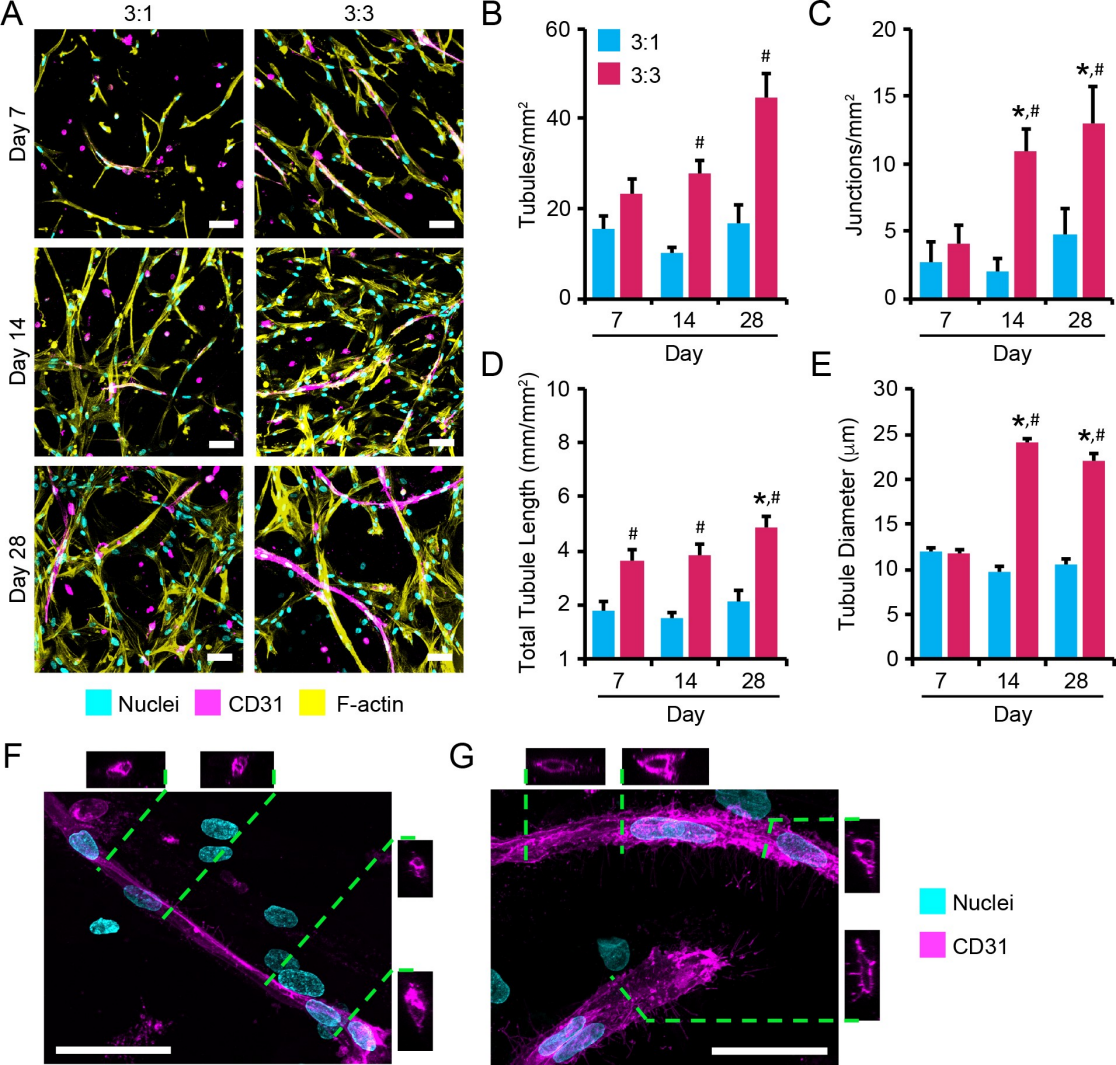
Assessment of MVEC microvessel formation in co-cultures. (**A**) Representative images of microvascular tubules in 3:1 and 3:3 MVEC:AoAF co-cultures in degradable 7.5wt% hydrogels with 3 mM RGD after 7, 14, and 28 days of culture. MVECs are depicted by CD31 (magenta), while F-actin is stained with phalloidin-568 (yellow) and nuclei are counterstained with Hoechst 33258 (cyan). Scale bar = 50 μm. (**B-F**) Quantification of network parameters including: (**B**) number of tubules, (**C**) number of junctions, (**D**) total tubule length, (**E**) tubule diameter. (**F-G**) Representative images MVEC microvessels in (**F**) 3:1 and (**G**) 3:3 co-cultures in 7.5wt% hydrogels with 3 mM RGD after 14 days of culture. Z-stack cross-sections demonstrate the formation of a hollow lumen structure in 3:3 co-cultures. MVECs are depicted by CD31 (magenta) and nuclei are counterstained with Hoechst 33258 (cyan). Scale bar = 50 μm. In **B-E**: data are represented as the mean ± SEM, with *n* = 3 biological replicates per condition. A two-way ANOVA, followed by a Tukey HSD post hoc test, was used to detect statistical significance, *p<0.05 for a given time point relative to day 7, within an individual co-culture, ^#^p<0.05 for 3:3 co-cultures compared to 3:1 co-cultures for a given time point.

To investigate associations between cell network formation observed in the degradable hydrogels and microvessel formation, and to evaluate whether the density of encapsulated AoAFs altered network and vessel formation in the two 3D hydrogel co-culture systems, MVEC networks were visualized over 28 days via immunofluorescent staining of CD31 (**Fig 3A**). CD31^+^ networks were observed in both 3:1 and 3:3 MVEC:AoAF co-cultures after 7 days and appeared to be stable throughout 28 days of culture. Quantification of MVEC network parameters revealed that both types of co-culture exhibited similar numbers of tubules (15.5 ± 2.7 versus 23.5 ± 3.1 tubules/mm^2^ for 3:1 and 3:3 cultures, respectively) after 7 days (**Fig 3B**). The number of tubules per mm^2^ increased significantly over time in the 3:3 co-cultures reaching 44.6 ± 5.4 tubules/mm^2^ after 28 days, while tubule number was not altered in the 3:1 co-cultures (16.7 ± 4.3 tubules/mm^2^ after 28 days). The number of multicellular network junctions did not differ significantly between the 3:1 and 3:3 co-cultures after 7 days, and a similar number of junctions (3:1: 2.7 ± 1.5 junctions/mm^2^; 3:3: 4.1 ± 1.4 junctions/mm^2^) were observed (**Fig 3C**). However, similar to the observed increase in tubules, the number of junctions increased significantly thereafter in 3:3 co-cultures (13.1 ± 2.7 junctions/mm^2^ after 28 days), but not in 3:1 co-cultures (4.8 ± 1.9 junctions/mm^2^ after 28 days). Interestingly, after 7 days of culture, 3:3 co-cultures also exhibited significantly longer tubules within projected culture areas (3.6 ± 0.4 mm/mm^2^) compared to 3:1 co-cultures (1.8 ± 0.4 mm/mm^2^; **Fig 3D**). Total tubule length in 3:3 co-culture increased over time, reaching 4.8 ± 0.4 mm/mm^2^ after 28 days. Conversely, total tubule length remained unchanged in 3:1 co-cultures over the 28 day time course (2.1 ± 0.4 mm/mm^2^). Average tubule diameters showed a similar pattern with initial diameters of 11.9 ± 0.6 μm and 11.7 ± 0.5 μm for 3:1 and 3:3 co-cultures, respectively (**Fig 3E**). No increase in average tubule diameter was observed for 3:1 co-cultures over time (10.6 ± 0.6 μm on day 28). However, the average tubule diameter increased to 24.0 ± 3.3 μm by 14 days in 3:3 co-cultures; vessel diameters were sustained between 14 and 28 days.

Lumen formation was assessed in the MVEC networks using slice views (x-z axis and y-z axis) obtained from 3D renderings of merged CD31 and Hoechst 33258 (nuclear) images. Lumenization was not observed for MVECs in 3:1 tubules at either 14 (**Fig 3F**) or 28 days (**S5 Fig**). However, MVECs in 3:3 co-cultures exhibited hollow structures after 14 days, as observed in cross-sectional images of microvessels, consistent with lumen formation within the nascent vascular structures (**Fig 3G**).

### AoAFs promote MVEC proliferation in 3D

**Fig 4:**
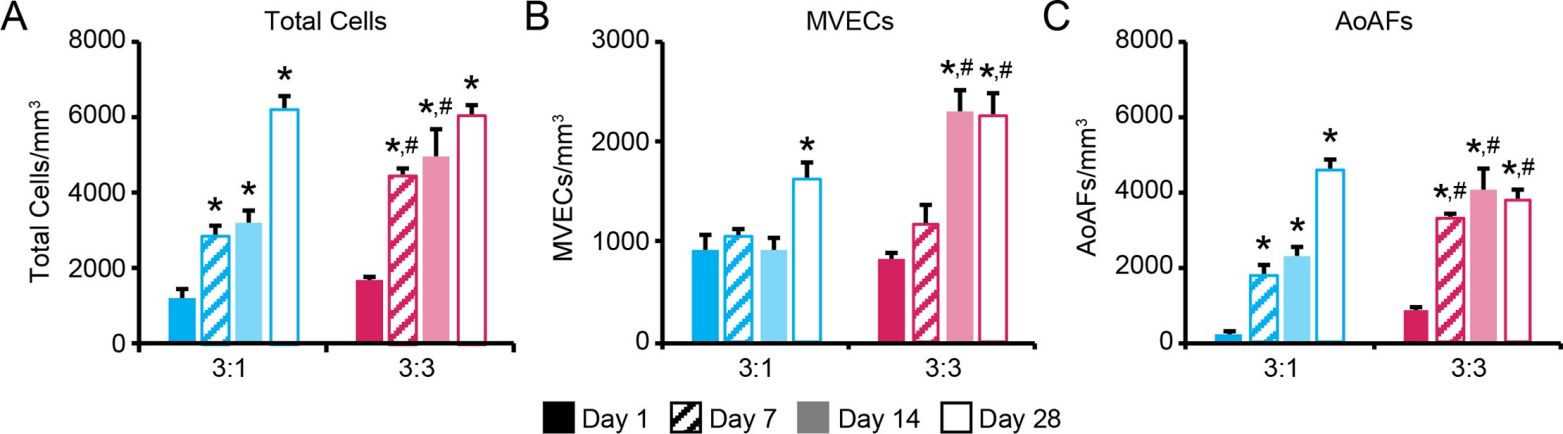
Assessment of proliferation in co-cultures. Number of (**A**) total cells, (**B**) MVECs, and (**C**) AoAFs per mm^3^ of degradable 7.5wt% hydrogel with 3 mM RGD in 3:1 and 3:3 MVEC: AoAF co-cultures over time. In **A-C**: a two-way ANOVA, followed by a Tukey HSD post hoc test, was used to detect statistical significance. *p<0.05 for a given time point relative to day 1, within an individual hydrogel formulation; ^#^p<0.05 for 3:3 co-cultures relative to 3:1 co-cultures at a given time point.

Adventitial fibroblasts have been shown to promote proliferation of vasa vasorum endothelial cells and the formation of microvessels both *in vitro* and *in vivo* [18]. Thus, we sought to determine whether the differences in network formation parameters observed in the 3:1 and 3:3 MVEC:AoAF co-cultures over time were associated with differences in cell proliferation. Cells were enumerated by identifying the total number of Hoechst 33258-stained nuclei in a defined hydrogel volume and extrapolating. Over the course of 28 days, 5.1- and 3.6-fold increases in total cell numbers were detected in the 3:1 and 3:3 hydrogel co-culture systems, respectively, indicating proliferation of encapsulated cells (**Fig 4A**). Although 3:3 co-cultures were initiated with 1.5-fold more cells than 3:1 co-cultures, a similar number of total cells was observed in both hydrogel systems after 28 days (3:1: 6203 ± 304 cells/mm^3^; 3:3: 6057 ± 289 cells/mm^3^).

To determine the relative contribution of MVECs and AoAFs to the populations of cells observed after 28 days, cells were enumerated separately by classifying CD31^+^ cells as MVECs and CD31^-^ cells as AoAFs. After an initial lag, the number of MVECs increased substantially in both the 3:1 and 3:3 co-culture systems (**Fig 4B**); however, temporal differences in MVEC proliferation were observed between the two co-culture systems. Significant increases in the number of MVECs in 3:3 co-cultures were observed after 14 days of culture, whereas significant MVEC proliferation was not observed until day 28 in the 3:1 co-cultures. Further, though both co-cultures were initiated with similar numbers of MVECs, and despite the overall elevated initial cell numbers, MVEC proliferation was higher in 3:3 co-cultures compared to 3:1 co-cultures after 28 days, such that MVECs had increased by 2.7-fold in 3:3 co-cultures, versus only a 1.8-fold increase in MVEC number was detected in 3:1 co-cultures.

AoAFs also proliferated in both 3:1 and 3:3 co-cultures over time, with significant increases in AoAF numbers observed in both co-culture systems after only 7 days of culture (**Fig 4C**). Although 3:1 co-cultures contained fewer AoAFs initially, by day 28, these co-cultures exhibited a similar number of AoAFs as the 3:3 hydrogels. Indeed, AoAF proliferation within PEG-based hydrogels was more pronounced in 3:1 co-cultures, where a 20.2-fold increase in the number of AoAFs was observed after 28 days, while only a 4.4-fold increase in AoAF number was detected in 3:3 hydrogel co-cultures. To determine whether AoAF proliferation in PEG-based hydrogels was dependent on the presence of MVECs, we initiated AoAF monocultures with cells at either 1x10^6^ AoAFs/mL hydrogel (0:1 monoculture) or 3x10^6^ AoAFs/mL hydrogel (0:3 monoculture). Increased AoAF proliferation observed over time in both co-culture systems correlated to proliferation trends in corresponding 0:3 and 0:1 monoculture systems (Pearson’s correlation: 3:3: R = 0.97, 3:1: 0.98; **S6 Fig**). However, the number of AoAFs in 3:3 co-cultures and 0:3 monocultures was similar over time (slope = 1.03), while AoAF proliferation in the 3:1 co-culture system was decreased compared to corresponding 0:1 monocultures (slope = 0.82).

### MVEC-AoAF contact results in formation of basement membrane proteins

**Fig 5:**
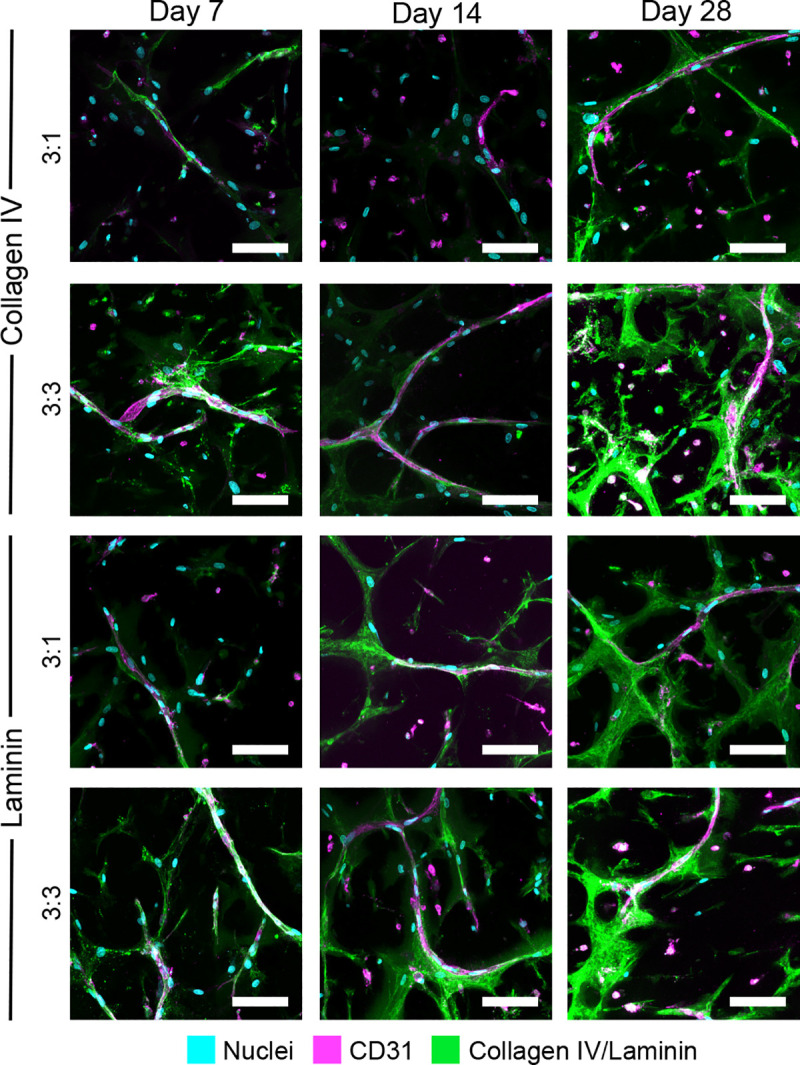
Production of collagen IV and laminin in co-cultures. Vessel-like structures in 3:3 MVEC:AoAF co-cultures, as well as 3:1 co-cultures, produce basal lamina proteins over time in degradable 7.5wt% hydrogels with 3 mM RGD. CD31^+^ MVEC networks (magenta) are surrounded by collagen type IV (green) or laminin (green). Nuclei are counterstained with Hoechst 33258 (blue). Scale bar = 100 μm.

Formation of the vascular basement membrane is a hallmark of vessel maturation [45]. In control cultures on 2D TCPS, MVECs and AoAFs were observed to independently produce both collagen IV and laminin (specifically laminins containing α1, α2, β1, β2, or γ3 subunits; **S7 Fig**). To next assess the capacity of the co-cultured MVEC and AoAF cells to secrete basement membrane proteins in 3D, we evaluated the production of collagen IV and laminin by AoAFs and MVECs in degradable hydrogels over 28 days via immunostaining (**Fig 5**). Collagen IV was present at low levels beginning after 7 days of culture in both the 3:1 and 3:3 MVEC:AoAF co-cultures with a predominant localization around the MVEC tubules in both systems. In the 3:3 co-cultures, collagen IV production increased over the 28-day period with localization around the MVEC tubules but also associated with AoAFs separated from tubules. In contrast, collagen IV production in 3:1 co-cultures remained low and predominantly localized around MVEC tubules throughout the duration of the study.

Laminin was also expressed at low levels after 7 days in both the 3:1 and 3:3 co-cultures. Laminin was predominantly localized to the MVEC tubules in 3:1 co-cultures at this early time point, while laminin in 3:3 co-cultures was detected both surrounding the MVEC tubules and in AoAFs that were not in contact with MVEC tubules. Laminin expression was observed to increase over time in both co-culture systems. After 28 days, laminin was observed to be localized around the MVEC tubules in both co-cultures and also present in AoAFs that were not next to the tubes.

### Direct cell contact is required for AoAF induction of MVEC tubules in PEG hydrogels

**Fig 6:**
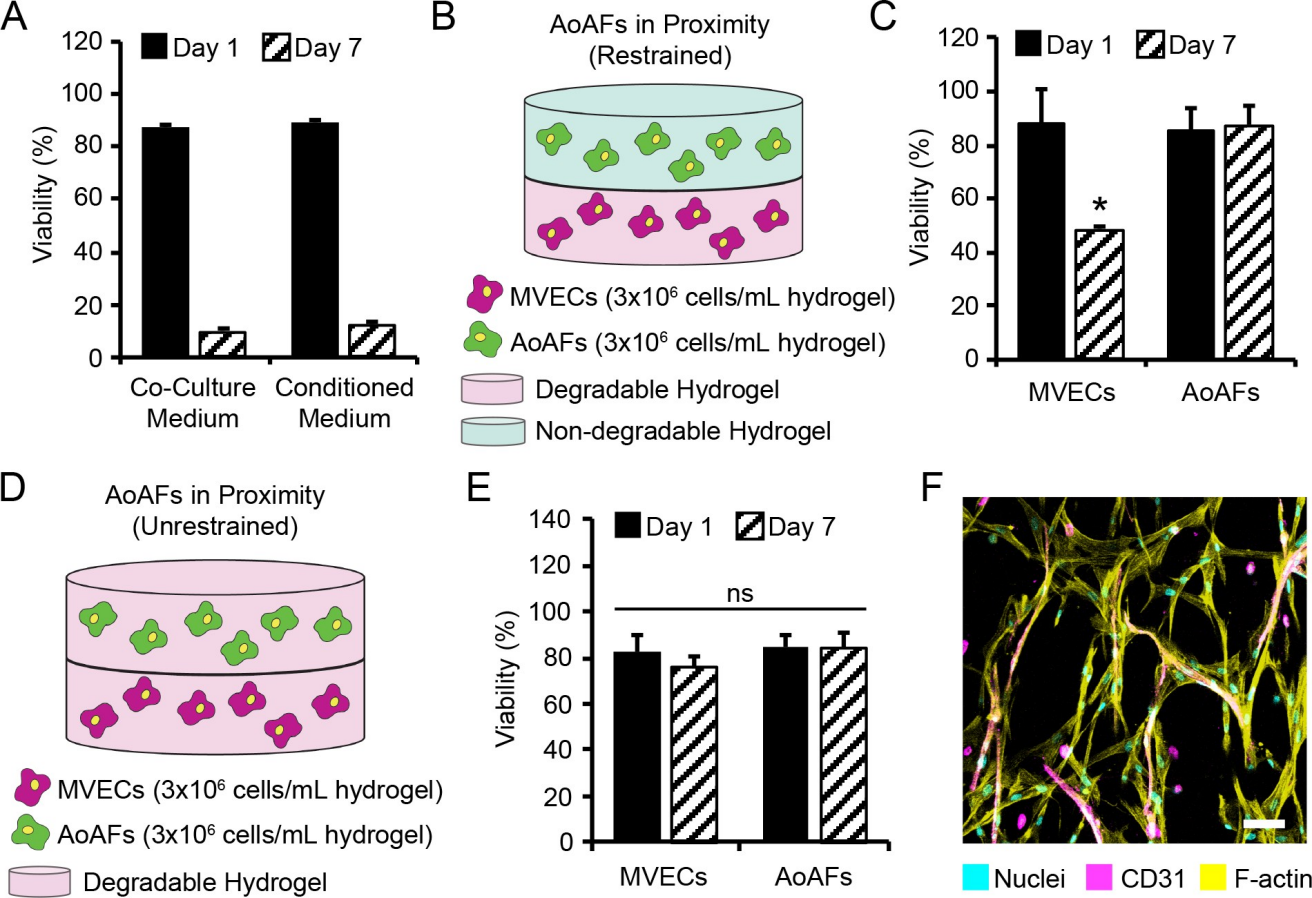
MVEC tubule formation requires direct contact with AoAFs. (**A**) To determine whether growth factors secreted by AoAFs promoted MVEC viability and spreading in degradable PEG hydrogels, encapsulated MVECs were cultured for 7 days in either fresh co-culture medium or AoAF conditioned medium. MVEC viability was high (>80%) after 1 day of encapsulation; however, viability significantly decreased after 7 days of culture in either fresh co-culture medium or AoAF conditioned medium. (**B**) To investigate whether direct contact between AoAFs and MVECs was necessary for directing microvessel formation, MVECs and AoAFs in separate layers of a hydrogel. MVECs were encapsulated in a layer of enzymatically degradable hydrogel (7.5wt% hydrogel with 3 mM RGD) and AoAFs were encapsulated in a layer of non-degradable hydrogel (10wt% hydrogel with 3 mM RGD) to limit direct contact of the two cell types. (**C**) Viability of encapsulated cells in the layered hydrogel system was high (>80%) after 24 hrs. AoAFs remained highly viable on day 7, while MVEC viability was significantly reduced. (**D**) However, when MVECs and AoAFs were encapsulated in separate layers of enzymatically degradable hydrogel, (**E**) MVEC viability was stable after 7 days of culture and (**F**) MVEC tubules formed at the intersection of degradable hydrogel layers after 14 days of culture. MVECs are depicted by CD31 (magenta), while F-actin is stained with phalloidin-568 (yellow) and nuclei are counterstained with Hoechst 33258 (cyan). Scale bar = 50 μm. In **A, C, & E**: data are represented as the mean ± SEM, with *n* = 3 biological replicates per condition. A two-way ANOVA, followed by a Tukey HSD post hoc test, was used to detect statistical significance, *p<0.05 for day 7 relative to day 1, within an individual cell system; ns = no significance.

Direct contact between mural cells and endothelial cells, as well as signaling between cells by diffusible factors, are considered critical for cell function and microvessel stability both *in vitro* and *in vivo* [46]. Accordingly, we evaluated the influence of MVEC-AoAF contacts on tubule formation in these PEG hydrogels. To determine the relative roles of growth factor signaling and cell-cell contact in AoAF-induced MVEC tubule formation in these hydrogel matrices, we examined tube formation by MVEC monocultures in degradable 3D hydrogels in response to AoAF-conditioned medium. High viability was maintained after 24 hrs of culture in either control medium or AoAF-conditioned media (87.2% ± 0.1% and 89.5% ± 0.9%, respectively; **Fig 6A and S8A Fig**). After 7 days, viability in MVEC monocultures was reduced, as in earlier studies, and not significantly altered by culture with AoAF conditioned medium reductions found to be similar to that of MVEC monocultures in control medium (26.4% ± 0.6% and 32.9% ± 0.9%, respectively).

As bulk dilution of growth factors in conditioned cell culture medium may result in an under-representation of local paracrine factor concentrations in vivo, we also performed experiments in which MVECs and AoAFs were cultured in close proximity, but direct cell-cell contact was impeded. To determine the impact of cell proximity on tubule formation in the absence of initial cell contact, a two-layer hydrogel system was employed. MVECs (3x10^6^ MVECs/mL) were encapsulated in a layer of enzymatically degradable PEG-based hydrogel, and a second layer of modulus matched, non-degradable PEG hydrogel containing encapsulated AoAFs was placed above (3x10^6^ AoAFs/mL; **Fig 6B**). The viability of encapsulated cells in the layered hydrogel system was high (>80%) after 24 hrs (**Fig 6C and S8B Fig**), and although little-to-no AoAF spreading was observed after 7 days of culture, AoAFs remained 85.1% ± 8.3% viable on day 7. Conversely, MVEC viability was significantly reduced by day 7 (48.0% ± 0.9%), with no apparent tube formation. Interestingly, when MVECs and AoAFs were encapsulated independently in layers of enzymatically degradable PEG hydrogels (**Fig 6D**), cell viability was maintained over time, such that 75.7% ± 5.2% of cells were viable on day 7 (**Fig 6E and S8C Fig**). Further, we observed spreading of the AoAFs in the degradable PEG hydrogels and the formation of MVEC tubules at the interface between the layered hydrogels after 14 days of culture (**Fig 6F**). Taken together, these results indicate that close proximity may be unimportant unless the two cell types are able to form direct AoAF-MVEC contacts that facilitate the growth of MVEC tubules in these PEG hydrogels.

### Phenotypic alterations in AoAFs observed during MVEC tubule stabilization

**Fig 7:**
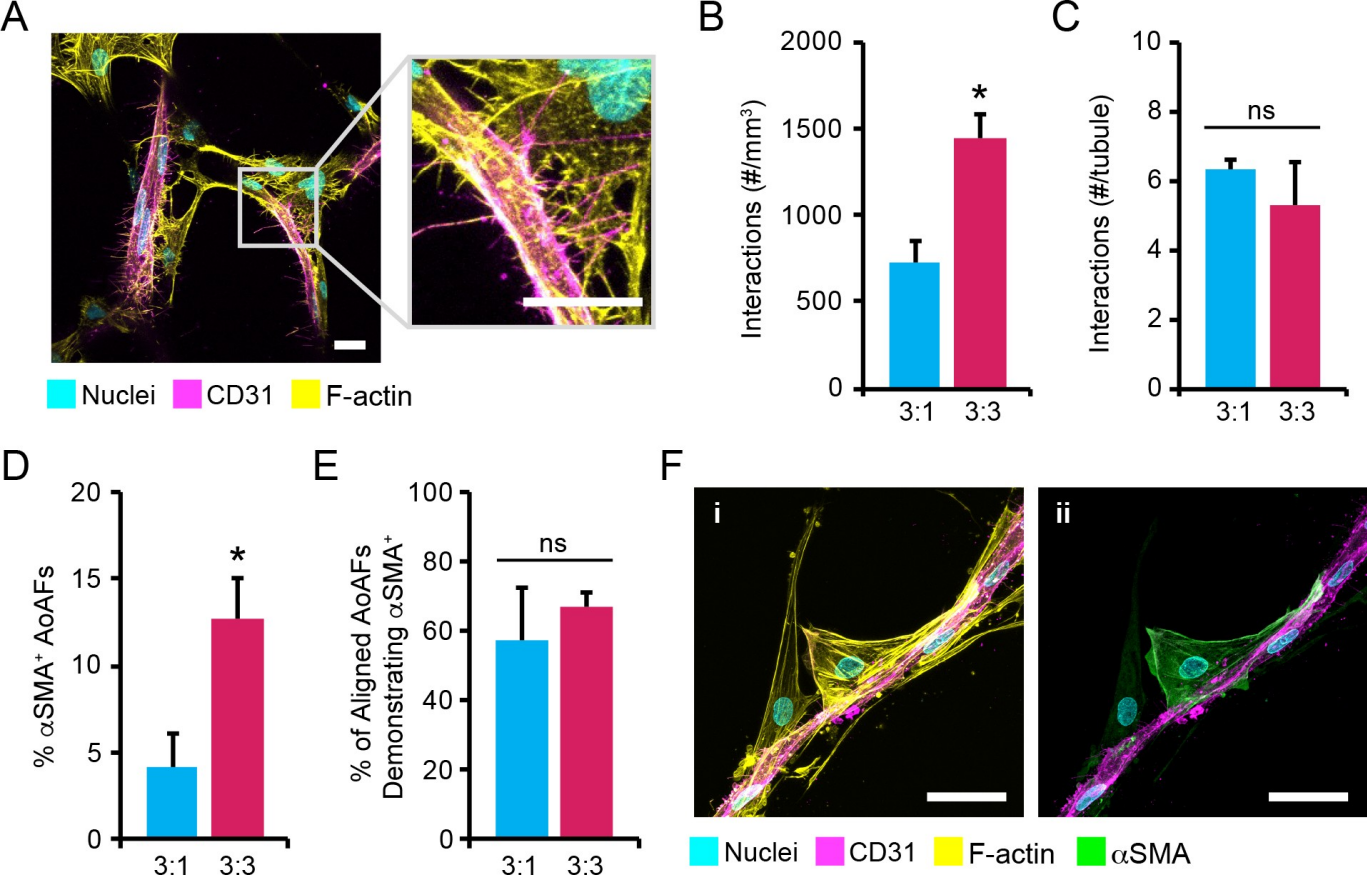
AoAF phenotype is altered following co-culture with MVECs. (**A**) Representative images of AoAFs directly interacting with MVECs in 3:3 MVEC:AoAF co-cultures in degradable 7.5wt% hydrogels with 3 mM RGD after 14 days of culture. MVECs are depicted by CD31 (magenta), while F-actin is stained with phalloidin-568 (yellow, AoAFs) and nuclei are counterstained with Hoechst 33258 (cyan). Scale bar = 20 μm. (**B-C**) Quantification of (**B**) the number AoAF interactions per mm^3^ and (**C**) the number AoAF interactions per tubule in the 3:1 and 3:3 co-cultures. (**D**) A low percentage of αSMA^+^ AoAFs were observed in 3:1 and 3:3 co-cultures after 14 days of co-culture. (**E**) However, the majority of AoAFs that were localized proximal to or directly adjacent to MVEC tubules were αSMA^+^ in both 3:1 and 3:3 co-cultures. (**F**) Representative images of αSMA^+^ AoAFs (green) aligned with CD31^+^ MVEC tubules (magenta) in 3:3 co-cultures on day 14. F-actin is stained with phalloidin-568 (yellow) and nuclei are counterstained with Hoechst 33258 (cyan). Scale bar = 50 μm. In **B-E**: data are represented as the mean ± SEM, with *n* = 3 biological replicates per condition. An unpaired student’s t-test detect statistical significance, *p<0.05 for 3:3 co-cultures relative to 3:1 co-cultures.

Cell interactions within our 3D degradable hydrogel networks were visualized after 14 days using CD31 to distinguish MVECs and visualization of F-actin networks to identify AoAFs (**Fig 7A**). Enumeration of AoAFs directly interacting with MVEC tubules demonstrated a significantly increased number of heterotypic cell-cell interactions in 3:3 MVEC:AoAF co-cultures (1441.1 ± 137.2 interactions/mm^3^) compared to 3:1 co-cultures (726.9 ± 122.1 interactions/mm^3^; **Fig 7B**). Interestingly, similar numbers of AoAFs interactions per MVEC tubule were observed in 3:1 and 3:3 co-cultures (3:3: 6.3 ± 1.9 interactions/tubule; 3:1: 5.3 ± 0.7 interactions/tubule; **Fig 7C**).

Mural cells typically arise from undifferentiated mesenchymal cells poised to become pericytes or SMCs following recruitment by emerging endothelial cell structures [26]. Thus, we investigated whether direct interactions between MVECs and AoAFs in degradable PEG-based hydrogels facilitated the transdifferentiation of AoAFs to a mural cell pericyte-like phenotype, which is typified by αSMA expression. Cultures were grown for 14 days in both the 3:1 and 3:3 systems then stained to detect CD31^+^ MVEC tubules and αSMA to identify AoAFs that differentiated towards a PC-like phenotype. 3D AoAF monocultures were used to establish baseline transdifferentiation rates, with 5.0% ± 1.8% of AoAFs were αSMA^+^ in monocultures after 14 days. Likewise, low levels of αSMA^+^ AoAFs (4.2% ± 2.0%) were observed in the 3:1 system after 14 days of culture (**Fig 7D**). The percentage of αSMA^+^ AoAFs in 3:3 co-cultures, on the other hand, was significantly higher, reaching 12.8% ± 2.3% αSMA^+^ AoAFs. Regardless, in both co-culture systems, the majority of AoAFs that were localized proximal to or directly adjacent to MVEC tubules were αSMA^+^ (3:1: 57.1% ± 15.2%; 3:3: 66.7% ± 4.4%) (**Fig 7E and 7F**).

### Impact of TGF-β signaling on vasculogenesis

Multiple studies show that TGF-β signaling promotes *de novo* vessel formation and recruitment of mural cell populations by ECs [25, 26]; however, the role of ALK5 is less understood. Thus, we sought to determine whether TGF-β signaling was involved in regulating MVEC viability and tube stability. We determined, via immunofluorescent staining, that both AoAFs and MVECs produced TGF-β1 and expressed the ALK5 receptor in standard 2D TCPS culture (**S9 Fig**). To determine whether AoAF and MVEC cultures actively utilize ALK5 mediated signaling pathways, cells were independently cultured with the ALK5 inhibitor, A83-01, in 2D. Inhibition of ALK5 using the inhibitor A83-01 reduced nuclear localization of SMAD-2/3 and increased vascular cell proliferation in standard 2D TCPS culture (**S10 Fig**), indicating that A83-01-sensitive pathways are active in both AoAF and MVEC cultures.

**Fig 8:**
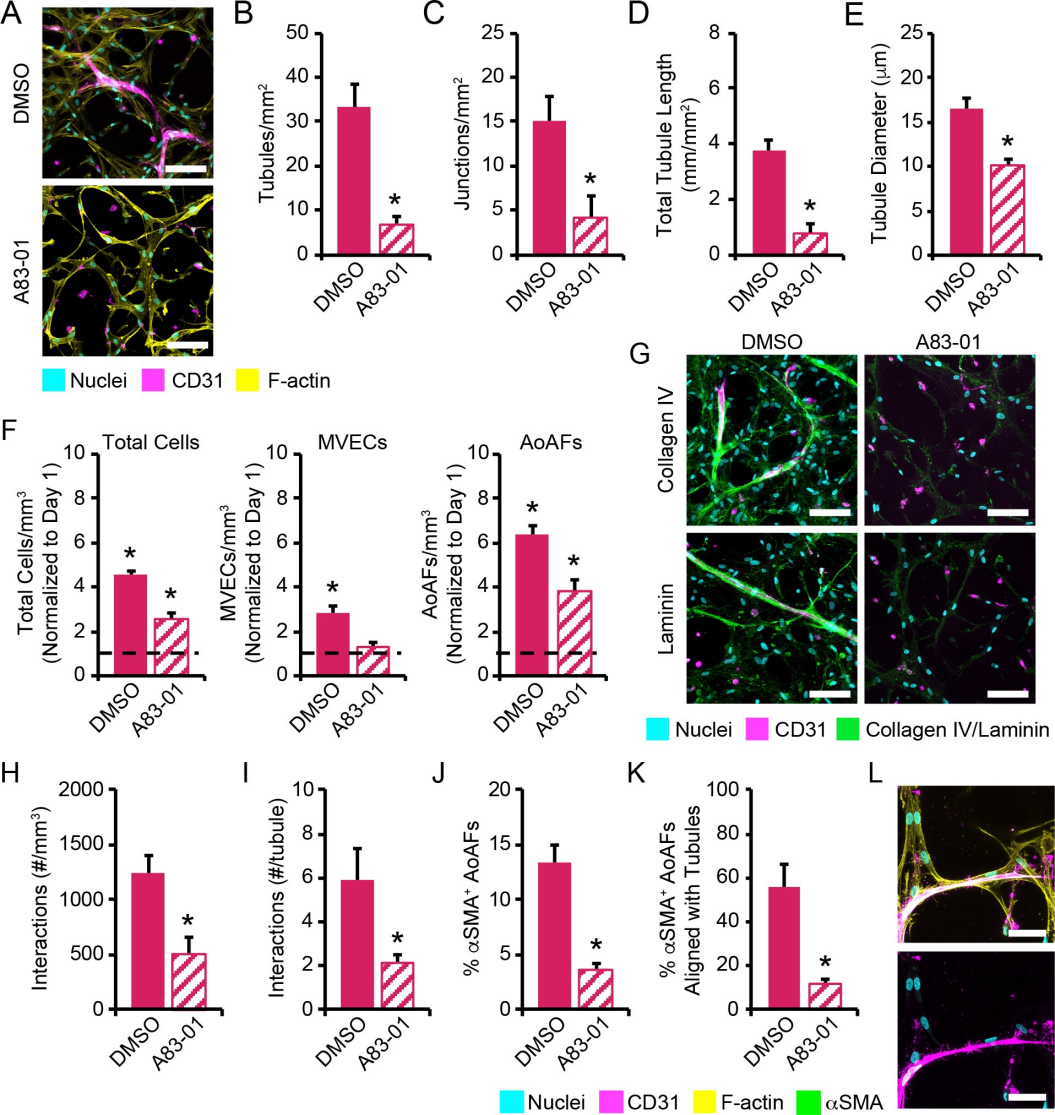
MVEC:AoAF interactions are dependent on signaling through ALK5. (**A**) Representative images of microvascular tubules in 3:3 MVEC:AoAF co-cultures in degradable 7.5wt% hydrogels with 3 mM RGD after 14 days of culture with 1 μM A83-01 (ALK5 inhibitor) or DMSO (control). MVECs are depicted by CD31 (magenta), while F-actin is stained with phalloidin-568 (yellow) and nuclei are counterstained with Hoechst 33258 (cyan). Scale bar = 100 μm. The addition of 1 μM A83-01 to 3:3 co-cultures resulted in significantly decreased (**B**) number of junctions per mm^2^, (**C**) number of MVEC tubules per mm^2^, (**D**) total tubule length (mm/mm^2^), (**E**) tubule diameter, compared to control co-cultures on day 14. (**F**) Following treatment with 1 μM A83-01, the number of total cells, MVECs, and AoAFs per mm^3^ was decreased compared to control cultures on day 14. Data are normalized to day 1 (dashed line). (**G**) Treatment with 1 μM A83-01 significantly reduced the production of the basal lamina proteins, collagen IV (green) and laminin (green), surrounding MVECs (magenta) compared to control co-cultures on day 14. Nuclei are counterstained with Hoechst 33258 (blue). Scale bar = 100 μm. Quantification of (**H**) the number AoAF interactions per mm^3^ and (**I**) the number AoAF interactions per tubule in co-cultures treated with 1 μM A83-01 or DMSO controls. (**J**) A low percentage of αSMA^+^ AoAFs were observed in 1 μM A83-01 co-cultures after 14 days and (**K**) were generally unaligned with MVEC tubules. (**L**) Representative images of αSMA^+^ AoAFs (green) in -cultures treated with 1 μM A83-01 on day 14. F-actin is stained with phalloidin-568 (yellow), nuclei are counterstained with Hoechst 33258 (cyan), and MVECs are labeled with CD31 (magenta). Scale bar = 50 μm. In **B-F, H-K**: data are represented as the mean ± SEM, with *n* = 3 biological replicates per condition. In **B-E, H-K**: an unpaired student’s t-test detect statistical significance, *p<0.05 for hydrogels treated with 1 μM A83-01 relative to control hydrogels. In **F**: a two-way ANOVA, followed by a Tukey HSD post hoc test, was used to detect statistical significance, *p<0.05 for hydrogels on day 14 relative day 1, ^#^p<0.05 for hydrogels treated with 1 μM A83-01 relative to control hydrogels on day 14.

To assess the role of TGF-β signaling in AoAF-mediated MVEC tubule formation in 3D, 3:3 MVEC:AoAF co-cultures in degradable hydrogels were treated with the ALK5 inhibitor A83-01 (1 μM) or DMSO (carrier control) for 14 days. Following culture, cell viability in A83-01-treated co-cultures was similar to that of controls (**S11 Fig**). MVEC network formation, visualized via immunofluorescent staining of CD31^+^ MVECs, was next evaluated and demonstrated that ALK5 inhibition reduced the formation of tubular structures in the co-cultures (**Fig 8A**). Quantification of the MVEC networks revealed that A83-01-treatment significantly decreased numbers of tubules (6.5 ± 1.4 tubules/mm^2^) compared to controls (33.3 ± 5.1 tubules/mm^2^) after 14 days (**Fig 8B**). Likewise, A83-01-treated co-cultures exhibited significant reductions in the number of junctions per mm^2^ (A83-01: 4.2 ± 2.5 junctions/mm^2^; DMSO: 15.0 ± 2.8 junctions/mm^2^), total tubule length (A83-01: 0.8 ± 0.3 mm/mm^2^; DMSO: 3.8 ± 0.3 mm/mm^2^), and average tubule diameter (A83-01: 16.6 ± 1.1 μm; DMSO: 10.2 ± 0.7 μm) compared to DMSO-treated controls (**Fig 8C–8E**). The reduction in microvessel formation in cultures treated with A83-01 corresponded to reduced total cell proliferation compared to DMSO-treated controls. Specifically, a 4.6-fold increase in total cell number was detected in DMSO controls after 14 days, while only a 2.6-fold increase was observed in A83-01 treated cultures (**Fig 8F**). When evaluated individually, MVECs exhibited decreased growth in A83-01 treated cultures compared to controls (A83-01: 1.3-fold increase from day 1; DMSO: 2.8-fold increase from day 1). Conversely, proliferation was observed for AoAFs A83-01 treated cultures compared to controls; however, AoAF numbers were significantly reduced compared to controls (A83-01: 3.8-fold increase from day 1; DMSO: 6.4-fold increase from day 1).

Treatment with A83-01 also significantly reduced the production of the basal lamina proteins, collagen IV and laminin, surrounding CD31^+^ MVEC networks in 3:3 co-cultures (**Fig 8G**). Cell interactions within our 3D hydrogel networks were visualized after 14 days using CD31 to distinguish MVECs and visualization of F-actin networks to identify AoAFs. Enumeration of AoAFs directly interacting with the MVEC tubules demonstrated a significantly reduced number of heterotypic cell-cell interactions in A83-01 treated co-cultures (503.2 ± 139.8 interactions/mm^3^, 2.1 ± 0.3 interactions/tubule) compared to controls (1247.8 ± 151.3 interactions/mm^3^, 5.9 ± 1.4 interactions/tubule; **Fig 8H and 8I**). Likewise, low levels of αSMA^+^ AoAFs (3.7% ± 0.4%) were observed in A83-01 treated co-cultures after 14 days of culture compared to controls (13.4% ± 1.6%; **Fig 8J**). In control co-cultures, the majority (58.4% ± 8.1%) of the αSMA^+^ AoAFs were localized proximal to or directly adjacent to MVEC tubules, while very few (11.4% ± 1.3%) αSMA^+^ AoAFs were localized proximal to or directly adjacent to MVEC tubules (**Fig 8K and 8L**).

## Discussion

Our co-culture studies using PEG hydrogels demonstrate that AoAFs critically regulate the formation of microvessels by enhancing MVEC viability, proliferation, and network formation. Further, inhibition of ALK5 signaling significantly reduced microvessel formation. Together, these findings suggest a critical role for adventitial fibroblasts in the formation of arterial vasa vasorum and indicate that interventions targeted at TGF-β pathways may alter the progression of vasa vasorum development. Regulation of vasa vasorum is critical for the healthy vascular function and the management of several types of vascular disease. Several groups have demonstrated the importance of AFs in regulating vasa vasorum development, homeostasis, and repair [16, 47]. However, elucidation of the specific cellular mechanisms involved in the formation of functional neovasculature has been difficult with current *in vivo* models. Our hydrogel platforms serve as dynamic and versatile *in vitro* models that afford insight into cellular interactions and were used to probe the fundamental mechanisms contributing to the formation of microvasculature. Further improvements in our understanding of AoAF cellular mechanisms involved in functional microvessel formation will allow for more precise application of current therapies and the development of new treatments.

A plethora of cell-binding molecules (e.g., fibrin, collagen, laminin, and oligopeptides) have been successfully incorporated into PEG-based hydrogels to promote EC tube-like structures [33, 48–50]. However, as we ultimately sought to investigate how MVEC-AoAF interactions resulted in neovascularization in this study, we first engineered a synthetic 3D matrix that supported vascular cells without directly initiating MVEC network formation in monocultures. Following evaluation of relevant biologically active laminin- and fibronectin-derived peptides on network formation in MVEC monocultures, hydrogels containing the cell adhesive ligand RGD were selected (**Fig 1, S2 and S3 Figs**), as these substrates did not initiate MVEC network formation and, therefore, could be used as a model system to study how MVEC-AoAF interactions support neovascularization. Although we observed a marked decrease in MVEC viability over time in the RGD-containing hydrogels (**Fig 1, S4 Fig**), this phenomenon was similarly observed for other matrices evaluated in this study and such decreases are consistent with previously reported behaviors of MVECs in 3D monoculture [51]. As we and others have utilized PEG-based materials to support growth of various vascular cell types in monoculture with a high degree of viability in each case [33, 34, 52], it is likely that MVECs simply require the support of mural cells, like AoAFs. Indeed, our studies unambiguously demonstrate the beneficial impact of AoAFs in sustaining MVEC viability and initiating cell spreading (**Fig 2**).

Our results demonstrate that AoAFs are capable of providing cell-contact-dependent signals to MVECs that drive the formation of stable EC networks (**Fig 3**). In particular, studies using AoAF-conditioned medium and co-culture studies in which AoAF were constrained in a separate non-degradable hydrogel layer both support the notion that cell contact is needed since neither condition facilitated MVEC tubule formation or stabilization of MVEC viability (**Fig 6A–6C**). We did observe that co-cultures consisting of MVECs and AoAFs embedded in separate but degradable hydrogel layers resulted in cell spreading and tube formation at the intersection of the layered hydrogels after 14 days of culture (**Fig 6D**). These findings are consistent with previous reports that direct heterotypic cell contact between SMCs and ECs is a requirement for the formation of microvessels [53]. Our work extends these observations to include AoAFs as heterotypic partner cells, and our observations suggest that the ability of separated AoAFs and MVECs to remodel the hydrogel network, potentially migrate, and initiate cell-cell contact is critical for microvessel formation. Indeed, vascularization *in vivo* is strongly associated with dynamic remodeling of cellular microenvironments [54]. We did observe that MVEC and AoAF monocultures both produced detectable MMP-2 (**S12 Fig**); however, MVEC monocultures produced MMP-2 at significantly lower levels than encapsulated AoAFs. Insufficient levels of active protease secretion in MVEC monocultures could result in an inability of the cells to remodel the hydrogel network and lead to the limited cell spreading observed in MVEC monocultures.

Microvessels were observed to form when MVECs were cultured directly with different relative densities of AoAFs; however, co-cultures with an equal MVEC-to-AoAF ratio (i.e. 3:3 co-cultures) yielded increased tubule formation, length, interconnectivity, and diameter compared to co-cultures containing a higher MVEC-to-AoAF ratio (i.e. 3:1 co-cultures; **Fig 3A–3E**), which is consistent with previous reports of interactions between MVECs and other ECs cultured with fibroblasts [44, 53]. Importantly, tissue engineered microvessels must contain lumen in order to anastomose with the host circulatory system [55], and characterization of the MVEC microvasculature structures in our studies revealed lumen formation in 3:3 co-culture systems (**Fig 3F**). Thus, formation of MVEC networks in our hydrogel system required only the presence of AoAFs, while maturation of neovascular tubes depended on the ratio of cell types present. In addition to manipulating cell ratios, alterations to the physicochemical properties of biomaterials can also serve as an effective mechanism for regulating microvessel formation [49, 56–59]. Intriguingly, our preliminary evaluation of the impact of hydrogel modulus and peptide concentration suggested that higher modulus and lower RGD concentration effectively altered vascular cell network measures (**S13 Fig**). However, additional studies are required to better understand microvascular cell response to varied matrix properties in this model system. Overall, these results suggest that simple manipulation of MVEC to mural cell ratio, as well as alteration to biomaterials properties, modulates vessel density and size in engineered structures.

Differences in the progression of microvessel growth within the two co-culture systems are likely due to altered rates of MVEC proliferation [60]. Indeed, MVEC expansion occurred in either co-culture only after a density of ~4000 AoAFs/mm^3^ was reached (**Fig 4**). Cultures initiated with a higher AoAF density (i.e. 3:3 co-cultures) reached this AoAF density requirement more quickly, resulting in enhanced MVEC proliferation and vessel growth at earlier time points. Our results suggest the existence of a critical threshold at which AoAF stimuli are sufficiently potent to initiate proliferation processes in MVECs, and corroborate previous findings that mural cells influence EC proliferation [61]. Interestingly, MVECs also influenced AoAF proliferation (**S6 Fig**), similar to previous reports [62]; however, this effect only occurred in cultures initiated with a higher MVEC-to-AoAF ratio, indicating that MVEC regulation of AoAF proliferation also occurs in a cell density-dependent manner. Interestingly, AoAFs cultured with MVECs exhibited decreased proliferation compared to AoAF monocultures initiated at the same density. Together, our results suggest that MVECs can restrict mural cell growth, as well as encourage it [46]. Though we only investigated the impact of the MVEC to AoAF ratio in 3D in this work, studies investigating the influence of cell ratio using MVECs and other stromal cell types would be of interest for tissue engineering approaches for generating microvasculature. Importantly, our results demonstrate that the general addition of AoAFs to the hydrogel promoted MVEC viability and microvessel formation in 3D.

Though AFs are often thought to be randomly distributed within the adventitial ECM, close cell-cell contacts do arise between endothelial cells and fibroblasts *in vivo* [51]. Indeed, fibroblast populations within the adventitia exhibit significant diversity, with reports suggesting that the AF population contains a subset MSC- or pericyte-like cells [63, 64], which localize alongside branching endothelial cells *in vitro* [18], consistent with other pericyte populations [65]. Though we did not specifically isolate adventitial-derived pericytes in this study, a portion of encapsulated AoAFs were observed to align adjacent to and be in direct contact with the tubular MVEC structures (**Fig 7A–7C**). Microvessel-adjacent AoAFs were observed to predominantly express αSMA, suggesting differentiation of these cells towards a pericyte/SMC phenotype (**Fig 7E**). Interestingly, not all AoAFs aligned adjacently to MVEC tubules and a portion of these unaligned cells also expressed αSMA, suggesting that the matrix itself stimulates transdifferentiation of a small percentage of AoAFs towards a myofibroblastic phenotype. Indeed, we have previously observed transdifferentiation of AoAFs into myofibroblasts in 3D PEG-based hydrogels [34, 36]. As pericyte populations share developmental origins with fibroblasts [63, 64] and are able to transition into myofibroblasts under pathological conditions [47], the expression of αSMA by both perivascular cells and myofibroblasts is not surprising. Future studies are needed to tease out the phenotypical and functional differences between AFs subpopulations in 3D biomaterials. However, perivascular localization of these αSMA cells, as well as deposition of basement membrane proteins, collagen IV and laminin (**Fig 5**), provide evidence of a stable microvascular phenotype in this PEG-based hydrogel system.

Details of the signaling pathways utilized by encapsulated AFs and MVECs provide insight into how cell-cell interactions guide neovascularization. TGF-β has been shown to induce fibroblast differentiation towards a pericyte phenotypes [66], as well as regulate EC proliferation and migration [35]. Recent reports have suggested that TGF-β-mediated activation and differentiation of ECs is mediated by two TGF‐β type I receptors, ALK1 and ALK5, with recent reports suggesting that ALK1 pathway activation turns on migratory and proliferative target genes in ECs, while increased activation of the ALK5 pathway results in inhibition of EC proliferation, migration, and vessel maturation [23]. Given the importance of ALK5 in regulating EC proliferation and microvessel formation, we investigated the role of TGF-β/ALK5 signaling in our 3D model system. Both MVECs and AoAFs produced TGF-β1 (**S9 Fig**), which obviated the need for exogenous TGF-β1, and inhibition of ALK5, via the pharmacological inhibitor A83-01, in MVEC monocultures on 2D TCPS resulted in expected promotion of cell growth (**S10 Fig**). However, ALK5 inhibition impeded both MVEC network formation and cell proliferation in our 3D co-cultures (**Fig 8A–8F**), indicating the central importance of this pathway in promoting tubule formation in our hydrogel system. Cell viability was not altered in co-cultures in response to ALK5 inhibition (**S11 Fig**), suggesting that MVEC survival is not regulated through the ALK5 signaling pathway and further indicating that cell death did not contribute to the observed reduction in tubule formation. Previous reports suggest that ALK5 signaling is required for ALK1-mediated EC proliferation and vessel morphogenesis [67]; thus, inhibition of this receptor in our 3D model system may have mitigated ALK1-mediated signaling processes in MVECs. Interestingly, we observed increased proliferation of MVEC monocultures on 2D TCPS, even following treatment with higher concentrations of the pharmacological ALK5 inhibitor A83-01 than those utilized in our 3D culture studies, suggesting that the role of ALK5 signaling in ECs may be context-dependent [68], and further emphasizing the complex nature of cell-cell interactions in neovessel formation.

In addition to regulating MVEC processes in neovasculogenesis, the ALK5 signaling pathway critically regulates mural cell recruitment [66]. Indeed, following ALK5 inhibition, MVECs did not appear to recruit AoAFs, and AoAFs appeared to be homogeneously distributed throughout inhibitor-treated hydrogels with very few MVEC-AoAF interactions observed (**Fig 8A**). Decreased mural cell recruitment, and consequently reduced MVEC network formation, in these cases may have stemmed from an inability of encapsulated cells to effectively remodel the hydrogel network following ALK5 inhibition. TGF-β1 is able to induce production of MMPs [69], and we found that ALK5 inhibition decreased MMP-2 production by encapsulated cells (**S14 Fig**), likely impeding matrix remodeling. Decreased collagen IV and total laminin production was also observed in A83-01-treated cultures (**Fig 8G**), underscoring the dependence of ECM production and remodeling in this system on the TGF-β/ALK5 signaling pathway.

## Conclusions

In conclusion, we demonstrated that AoAFs critically regulate neovasculogenesis in a 3D hydrogel model system. Utilizing PEG-based substrates, which served as a passive scaffold to study MVEC-AoAF interactions, we determined that AoAF density, as well as the physiochemical properties of the hydrogel platform, regulate microvessel formation, while microvessel stabilization was dependent on perivascular recruitment of αSMA^+^ AoAFs and the deposition of basement membrane proteins. We further showed that while ALK5 does not regulate MVEC survival, this receptor does mediate microvessel formation, as inhibition of the ALK5 signaling pathway significantly reduced MVEC proliferation, microvessel formation, mural cell recruitment, basement membrane production. These results emphasize the complicated and robust mechanisms associated with cell-cell signaling pathways relevant to neovasculogenesis. Our data suggests that therapeutics targeting the TGF-β/ALK5 pathway may be useful for regulation of vasculogenic and anti-vasculogenic responses *in vivo*. Because of the expression of ALK5 on both MVECs and AoAFs, we were not able determine the relative contributions of ALK5 signaling for the individual cell types in the present work. Future work that focuses on cell-specific responses, including evaluation of the individual impact of ALK5 signaling on MVEC and AoAF activities, as well as whether the effects observed here are due to autocrine or paracrine bidirectional signaling, will be necessary. Additional studies resolving the relationships between signaling pathways, cell-cell interactions, and cell behavior will be critical to advance our understanding of neovascularization, particularly in the context of arterial disease and the development of advanced therapies.

## Supporting information

S1 Fig Co-culture medium supports proliferation of MVECs and AoAFs in 2D. Proliferation of AoAFs and MVECs following culture in native medium (AoAFs: FGM; MVECs: EGM2-MV) or co-culture medium (1:1 EGM2-MV:FGM) for 3 days. Data are normalized to day 0 (dashed line). Data are represented as the mean ± SEM, with *n* = 3 biological replicates per condition. A repeated measures two-way ANOVA, followed by a Tukey HSD post hoc test, was used to detect statistical significance, *p<0.05 for day 3 relative to day 0, within an individual cell type/medium combination, ^#^p<0.05 for cells treated with co-culture medium relative to cells treated with native medium on day 3.
(TIF)

S2 Fig Laminin-based peptides influence MVEC tube formation on Matrigel. Tube formation assay. MVECs were seeded on Matrigel with and without laminin-derived peptides. All laminin peptides were introduced at a concentration of 0.2 mM. Reduced MVEC tube formation was observed after 4 hrs in the presence of three laminin-derived peptides, AG10, AG73, and YIGSR. The A2G78 peptide did not have a significant effect. Scale bar = 500 μm.
(TIF)

S3 Fig MVECs attach to PEG hydrogels containing RGD peptides. Representative images of MVECs cultured on non-degradable PEG hydrogels containing 0 or 1 mM of RGD-MI peptide for 24 hrs. MVECs cultured on hydrogels containing 1 mM RGD were adherent. Hydrogels lacking RGD did not promote cell adhesion, with MVECs observed to be rounded and clustered. Scale bar = 200 μm.
(TIF)

S4 Fig MVEC viability is decreased over time in PEG-based hydrogels. Representative live/dead images MVEC monocultures in 7.5wt% hydrogels with 3 mM RGD, 3 mM YIGSR, and 3 mM laminin peptide cocktail after 1 and 7 days of culture. Green indicates live cell bodies and red indicates nuclei in necrotic cells. Scale bar = 100 μm.
(TIF)

S5 Fig Lumen structure formation in co-cultures after 28 days. Representative images MVEC microvessels in (**A**) 3:1 and (**B**) 3:3 co-cultures in 7.5wt% hydrogels with 3 mM RGD after 28 days of culture. Z-stack cross-sections demonstrate the formation of a hollow lumen structure in 3:3 co-cultures. MVECs are depicted by CD31 (magenta) and nuclei are counterstained with Hoechst 33258 (cyan). Scale bar = 50 μm.
(TIF)

S6 Fig AoAF proliferate in both mono- and co-cultures. Comparison of the number of AoAFs per mm^3^ in (**A**) 3:3 co-cultures vs. 0:3 AoAF monocultures and (**B**) 3:1 co-cultures vs. 0:1 AoAF monocultures over time in 7.5wt% hydrogel with 3 mM RGD.
(TIF)

S7 Fig AoAFs and MVECs secrete basement membrane proteins in 2D culture. Monocultures of MVECs and AoAFs produce collagen type IV (green) and laminin (green) on TCPS. F-actin is stained with phalloidin-568 (gray) and nuclei are counterstained with Hoechst 33258 (blue). Scale bar = 100 μm.
(TIF)

S8 Fig Viability of MVECs is impaired when direct contact with AoAFs is restricted. (**A**) Representative live/dead images of MVECs in degradable PEG-based hydrogel (7.5wt% hydrogels with 3 mM RGD) cultured in either co-culture medium (1:1 EGM2-MV) or AoAF conditioned medium. (**B**) Representative live/dead images of MVECs and AoAFs in the restricted layered hydrogel system after 1 and 7 days of culture. MVECs were encapsulated in a layer of degradable PEG-based hydrogel (7.5wt% hydrogels with 3 mM RGD), while AoAFs were encapsulated in a layer of non-degradable PEG-based hydrogel (10wt% hydrogels with 3 mM RGD). (**C**) In the unrestricted layered hydrogel system, MVECs were first encapsulated in a layer of degradable PEG-based hydrogel, followed by encapsulation of AoAFs in a second layer of degradable PEG-based hydrogels. Green indicates live cell bodies and red indicates nuclei in necrotic cells. Scale bar = 100 μm.
(TIF)

S9 Fig AoAFs and MVECs express TGF-β1 and ALK5. Representative images of TGF-β1 (green) and ALK5 (green) in AoAFs and MVECs cultured on TCPS. F-actin is stained with phalloidin-568 (red) and nuclei are counterstained with Hoechst 33258 (blue). Scale bar = 100 μm.
(TIF)

S10 Fig Inhibition of ALK5 reduces the nuclear localization of SMAD-2/3 while increasing cell proliferation. (**A**) Representative images of SMAD-2/3 (green) localization in AoAFs and MVECs cultured on TCPS, following treatment with either 0.5 μM A83-01 (ALK5 inhibitor) or DMSO (control) for 72 hrs. F-actin is stained with phalloidin-568 (red) and nuclei are counterstained with Hoechst 33258 (blue). Scale bar = 100 μm. (**B**) Normalized AoAF and MVEC proliferation following treatment with either 0.5 μM or 5 μM A83-01 (ALK5 inhibitor) or DMSO (control) for 72 h. Data are normalized to day 0 (dashed line). In **B:** Data are represented as the mean ± SEM, with *n* = 3 biological replicates per condition. A repeated measures two-way ANOVA, followed by a Tukey HSD post hoc test, was used to detect statistical significance, *p<0.05 for day 3 relative to day 0, within an individual cell type/inhibitor combination, ^#^p<0.05 for A83-01-treated cultures relative to controls on day 3.
(TIF)

S11 Fig Vascular cell viability is not altered following ALK5 inhibition. (**A**) Representative live/dead images of 3:3 MVEC:AoAF co-cultures in 7.5wt% hydrogels with 3 mM RGD after 1 and 7 days of culture with 1 μM A83-01 or DMSO (control). Green indicates live cell bodies and red indicates nuclei in necrotic cells. Scale bar = 100 μm. (**B**) Over 85% viability was observed for vascular cells encapsulated PEG hydrogels after 1 day of treatment with 1 μM A83-01 or DMSO (control). High viability was maintained (>85%) in 3:3 co-cultures even after 7 days of treatment with 1 μM A83-01 or DMSO (control). In **B**: data are represented as the mean ± SEM, with *n* = 3 biological replicates per condition. A repeated measures one-way ANOVA was used to detect statistical significance.
(TIF)

S12 Fig AoAFs secrete increased MMP-2 levels compared to MVECs. Encapsulated AoAFs produced significantly more MMP‐2, as detected via ELISA, compared encapsulated MVECs. MVECs and AoAFs were encapsulated independently at 3x10^6^ cells/mL of 7.5wt% hydrogel containing 3 mM RGD. Conditioned medium was collected every 3 days and stored at -80°C until analysis. Data are represented as the mean ± SEM, with *n* = 3 biological replicates per condition. A repeated measures two-way ANOVA, followed by a Tukey HSD post hoc test, was used to detect statistical significance, *p<0.05 for AoAFs relative to MVECs.
(TIF)

S13 Fig MVEC microvessel formation is restricted in hydrogels exhibiting increased stiffness or decreased concentration of adhesive peptides. (**A**) The initial equilibrium storage moduli (G’, kPa), evaluated by oscillatory shear rheology after 24 h, significantly increased with increasing wt%. (**B**) Representative images of microvascular tubules in 7.5wt% and 15wt% hydrogels after 14 days of culture. MVECs are depicted by CD31 (magenta), while F-actin is stained with phalloidin-568 (yellow) and nuclei are counterstained with Hoechst 33258 (blue). Scale bar = 100 μm. Increasing hydrogel wt% did not impact (**C**) the number of junctions per mm^2^ or the (**D**) the number of MVEC tubules per mm^2^ after 14 days of culture. (**E**) Total tubule length (mm/mm^2^) and (**F**) tubule diameter decreased significantly as hydrogel wt% increased. (**G**) The initial equilibrium storage moduli (G’, kPa), evaluated by oscillatory shear rheology after 24 h, was similar for 7.5wt% hydrogels containing either 1 mM or 3 mM RGD. (**H**) Representative images of microvascular tubules in 7.5wt% hydrogels with 1 or 3 mM RGD after 14 days of culture. MVECs are depicted by CD31 (magenta), while F-actin is stained with phalloidin-568 (yellow) and nuclei are counterstained with Hoechst 33258 (blue). Scale bar = 100 μm. (**I**) RGD concentration did not impact the number of junctions per mm^2^ between MVEC tubules. (**J**) The number of tubules per mm^2^ and (**K**) total tubule length (mm/mm^2^) increased significantly with increasing RGD concentration after 14 days of culture. (**L**) Tubule diameter on day 14 significantly increased with increasing RGD concentration. In **A, C-G, I-L**: data are represented as the mean ± SEM, with *n* = 3 biological replicates per condition. An unpaired student’s t-test detect statistical significance, *p<0.05 for 7.5 wt% hydrogels containing 3 mM RGD relative to either (**A, C-F**) 15wt% hydrogels containing 3 mM RGD or (**G, I-L**) 7.5wt% hydrogels containing 1 mM RGD.
(TIF)

S14 Fig MMP-2 production is decreased following ALK5 inhibition. MMP-2 production was decreased in co-cultures treated with 1 μM A83-01 compared to DMSO-treated controls, as detected via ELISA. Data are represented as the mean ± SEM, with *n* = 3 biological replicates per condition. A repeated measures two-way ANOVA, followed by a Tukey HSD post hoc test, was used to detect statistical significance, *p<0.05 for control cultures relative to cultures treated with 1 μM A83-01, at a given timepoint.
(TIF)

S1 Table Antibodies used for immunostaining of MVECs and AoAFs. (DOCX)

S2 Table Cell adhesion peptides used in laminin-derived peptide hydrogels. (DOCX)

S1 File Supplemental methods. A description of all supplemental methods.
(PDF)
