# Supplementary figures and images for: Regulation of neovasculogenesis in co-cultures of aortic adventitial fibroblasts and microvascular endothelial cells by cell-cell interactions and TGF-β/ALK5 signaling
Source: PLoS One. 2020 Dec 28;15(12):e0244243. doi: 10.1371/journal.pone.0244243 (PMC7769260; doi:10.1371/journal.pone.0244243)

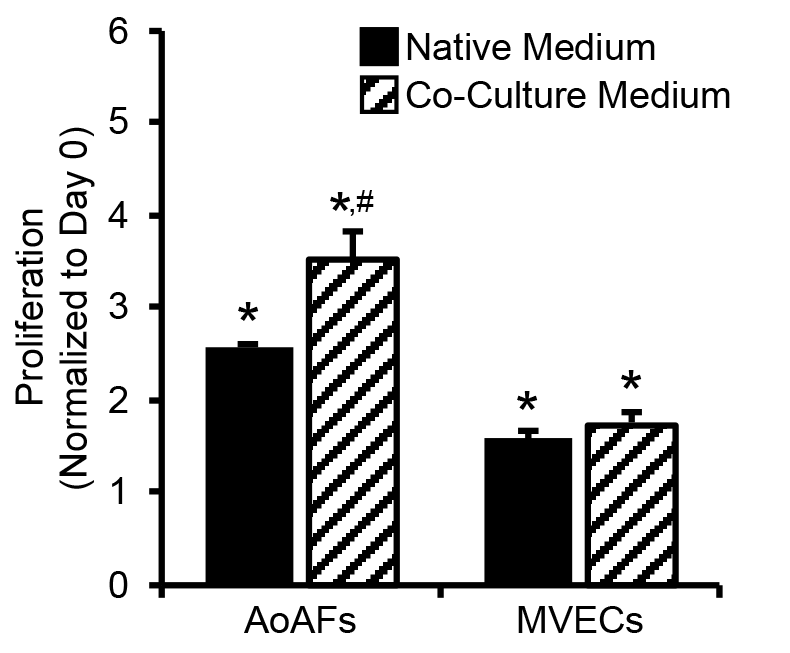

Supplement: S1 Fig — Proliferation of AoAFs and MVECs following culture in native medium (AoAFs: FGM; MVECs: EGM2-MV) or co-culture medium (1:1 EGM2-MV:FGM) for 3 days. Data are normalized to day 0 (dashed line). Data are represented as the mean ± SEM, with n = 3 biological replicates per condition. A repeated measures two-way ANOVA, followed by a Tukey HSD post hoc test, was used to detect statistical significance, *p<0.05 for day 3 relative to day 0, within an individual cell type/medium combination, #p<0.05 for cells treated with co-culture medium relative to cells treated with native medium on day 3. (TIF) [file pone.0244243.s001.tif]

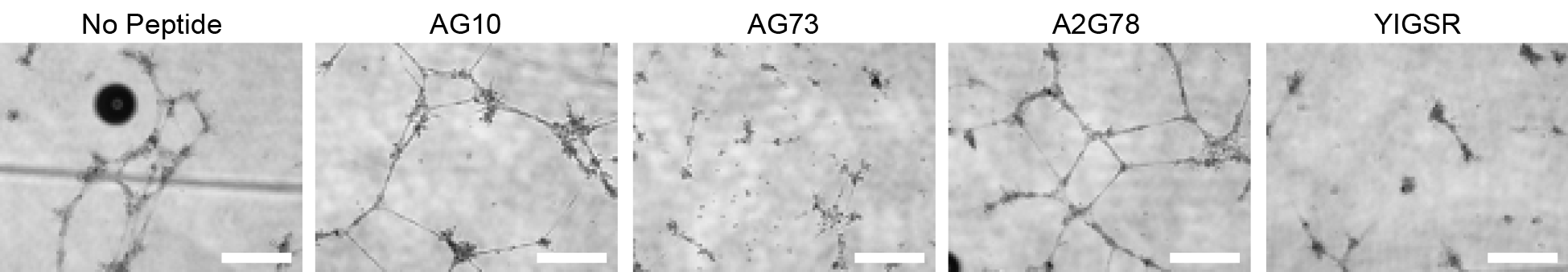

Supplement: S2 Fig — Tube formation assay. MVECs were seeded on Matrigel with and without laminin-derived peptides. All laminin peptides were introduced at a concentration of 0.2 mM. Reduced MVEC tube formation was observed after 4 hrs in the presence of three laminin-derived peptides, AG10, AG73, and YIGSR. The A2G78 peptide did not have a significant effect. Scale bar = 500 μm. (TIF) [file pone.0244243.s002.tif]

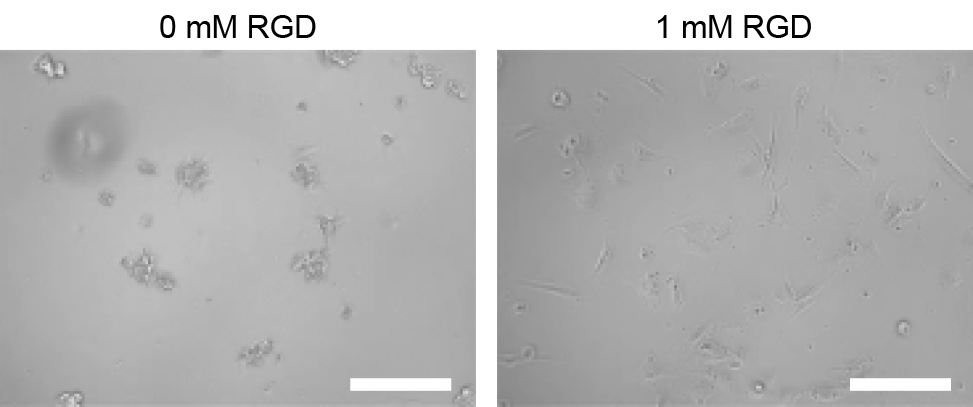

Supplement: S3 Fig — Representative images of MVECs cultured on non-degradable PEG hydrogels containing 0 or 1 mM of RGD-MI peptide for 24 hrs. MVECs cultured on hydrogels containing 1 mM RGD were adherent. Hydrogels lacking RGD did not promote cell adhesion, with MVECs observed to be rounded and clustered. Scale bar = 200 μm. (TIF) [file pone.0244243.s003.tif]

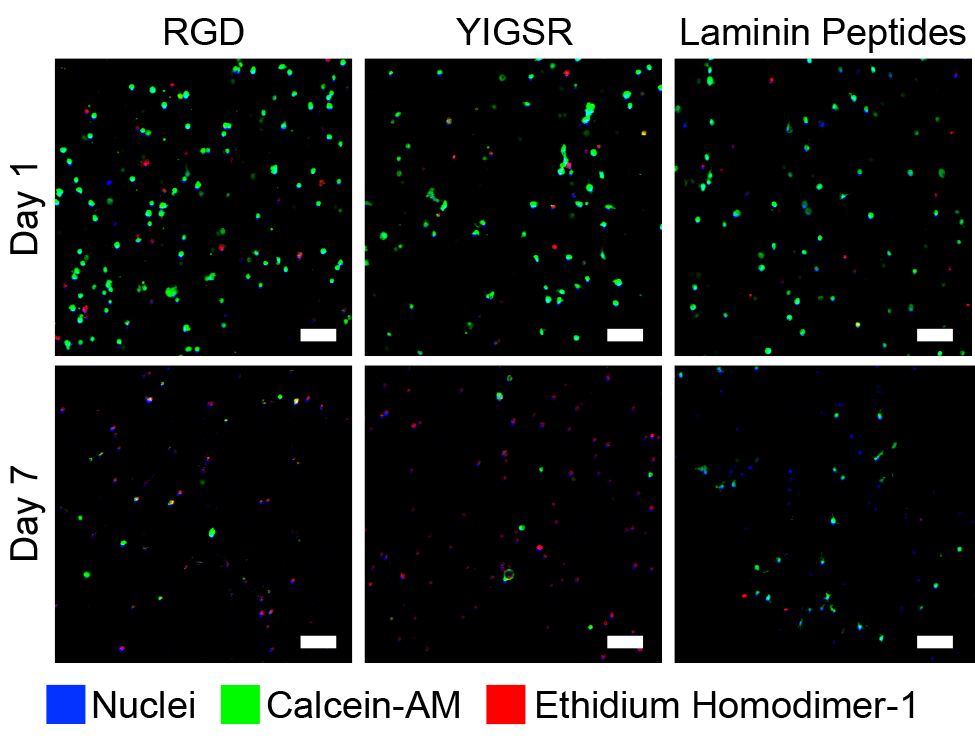

Supplement: S4 Fig — Representative live/dead images MVEC monocultures in 7.5wt% hydrogels with 3 mM RGD, 3 mM YIGSR, and 3 mM laminin peptide cocktail after 1 and 7 days of culture. Green indicates live cell bodies and red indicates nuclei in necrotic cells. Scale bar = 100 μm. (TIF) [file pone.0244243.s004.tif]

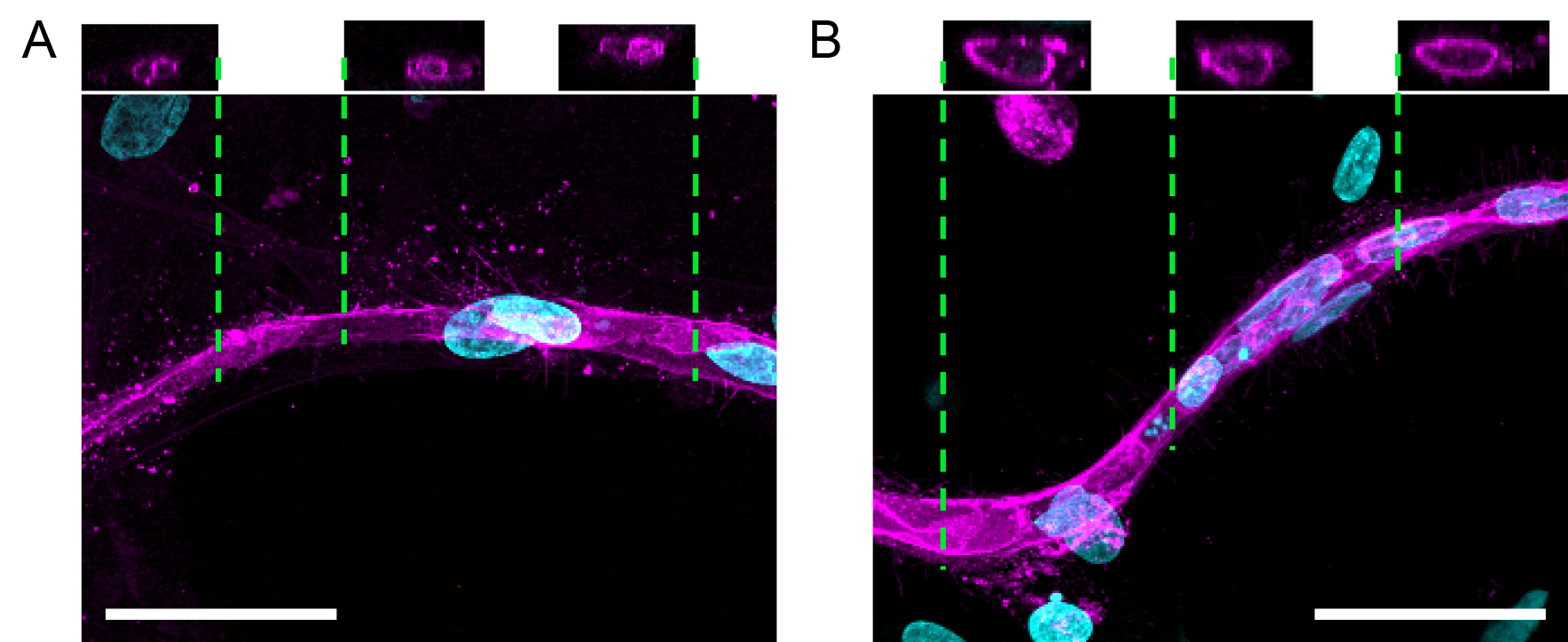

Supplement: S5 Fig — Representative images MVEC microvessels in (A) 3:1 and (B) 3:3 co-cultures in 7.5wt% hydrogels with 3 mM RGD after 28 days of culture. Z-stack cross-sections demonstrate the formation of a hollow lumen structure in 3:3 co-cultures. MVECs are depicted by CD31 (magenta) and nuclei are counterstained with Hoechst 33258 (cyan). Scale bar = 50 μm. (TIF) [file pone.0244243.s005.tif]

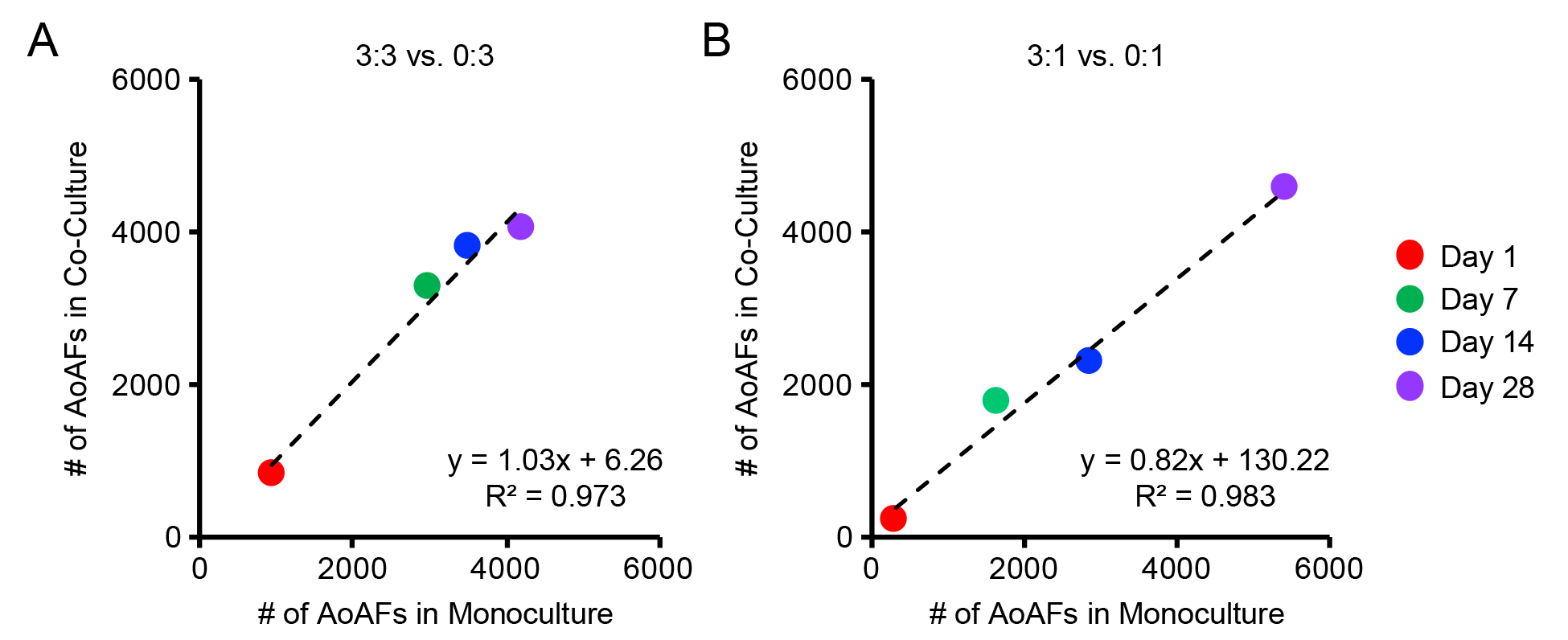

Supplement: S6 Fig — Comparison of the number of AoAFs per mm3 in (A) 3:3 co-cultures vs. 0:3 AoAF monocultures and (B) 3:1 co-cultures vs. 0:1 AoAF monocultures over time in 7.5wt% hydrogel with 3 mM RGD. (TIF) [file pone.0244243.s006.tif]

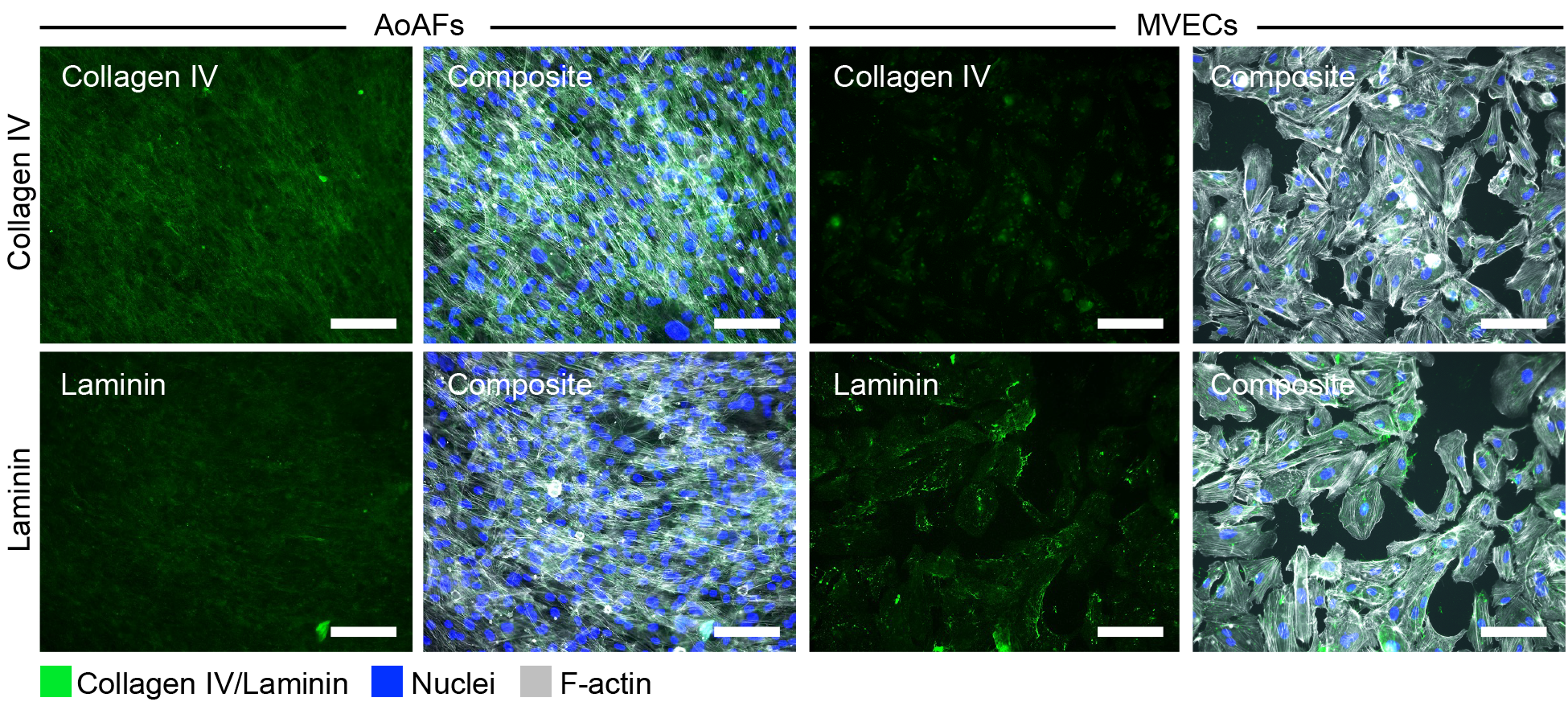

Supplement: S7 Fig — Monocultures of MVECs and AoAFs produce collagen type IV (green) and laminin (green) on TCPS. F-actin is stained with phalloidin-568 (gray) and nuclei are counterstained with Hoechst 33258 (blue). Scale bar = 100 μm. (TIF) [file pone.0244243.s007.tif]

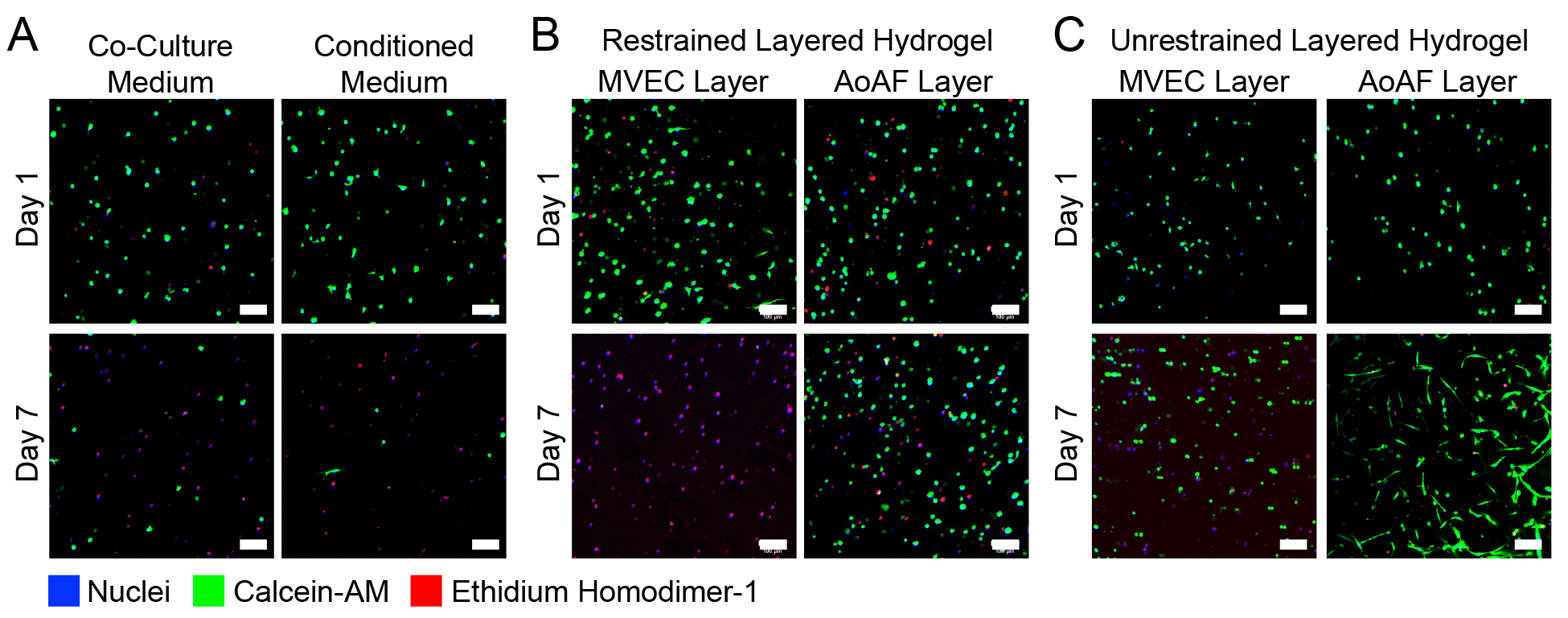

Supplement: S8 Fig — (A) Representative live/dead images of MVECs in degradable PEG-based hydrogel (7.5wt% hydrogels with 3 mM RGD) cultured in either co-culture medium (1:1 EGM2-MV) or AoAF conditioned medium. (B) Representative live/dead images of MVECs and AoAFs in the restricted layered hydrogel system after 1 and 7 days of culture. MVECs were encapsulated in a layer of degradable PEG-based hydrogel (7.5wt% hydrogels with 3 mM RGD), while AoAFs were encapsulated in a layer of non-degradable PEG-based hydrogel (10wt% hydrogels with 3 mM RGD). (C) In the unrestricted layered hydrogel system, MVECs were first encapsulated in a layer of degradable PEG-based hydrogel, followed by encapsulation of AoAFs in a second layer of degradable PEG-based hydrogels. Green indicates live cell bodies and red indicates nuclei in necrotic cells. Scale bar = 100 μm. (TIF) [file pone.0244243.s008.tif]

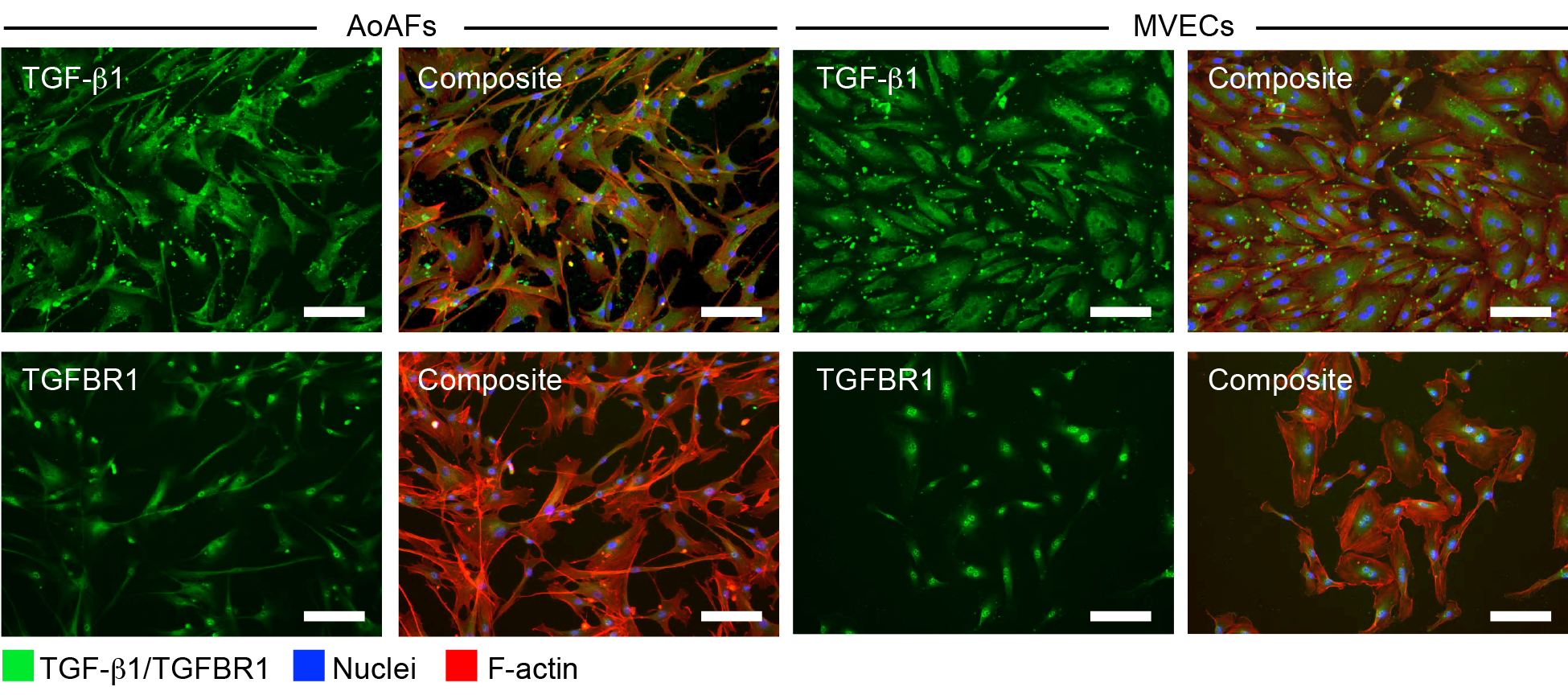

Supplement: S9 Fig — Representative images of TGF-β1 (green) and ALK5 (green) in AoAFs and MVECs cultured on TCPS. F-actin is stained with phalloidin-568 (red) and nuclei are counterstained with Hoechst 33258 (blue). Scale bar = 100 μm. (TIF) [file pone.0244243.s009.tif]

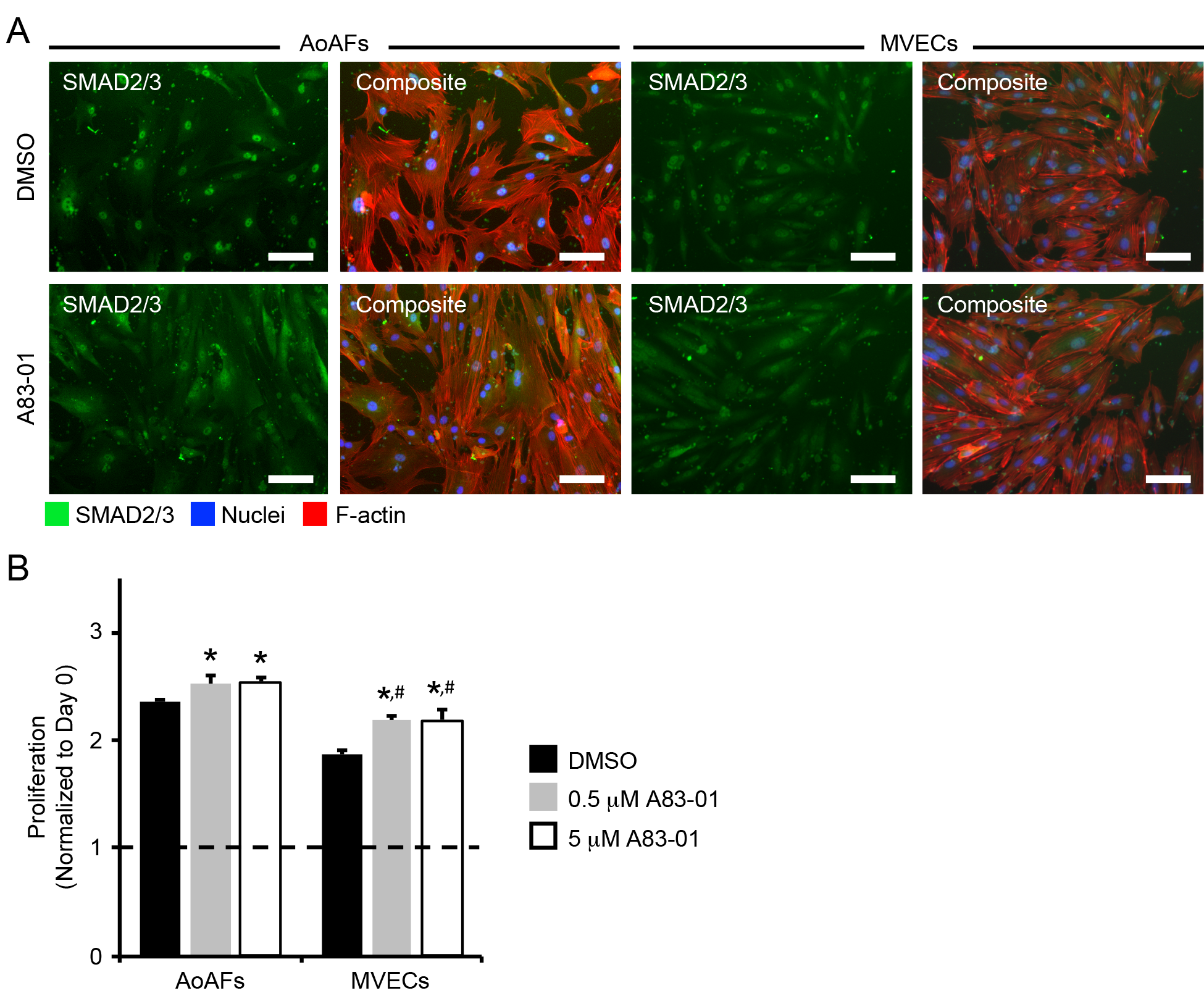

Supplement: S10 Fig — (A) Representative images of SMAD-2/3 (green) localization in AoAFs and MVECs cultured on TCPS, following treatment with either 0.5 μM A83-01 (ALK5 inhibitor) or DMSO (control) for 72 hrs. F-actin is stained with phalloidin-568 (red) and nuclei are counterstained with Hoechst 33258 (blue). Scale bar = 100 μm. (B) Normalized AoAF and MVEC proliferation following treatment with either 0.5 μM or 5 μM A83-01 (ALK5 inhibitor) or DMSO (control) for 72 h. Data are normalized to day 0 (dashed line). In B: Data are represented as the mean ± SEM, with n = 3 biological replicates per condition. A repeated measures two-way ANOVA, followed by a Tukey HSD post hoc test, was used to detect statistical significance, *p<0.05 for day 3 relative to day 0, within an individual cell type/inhibitor combination, #p<0.05 for A83-01-treated cultures relative to controls on day 3. (TIF) [file pone.0244243.s010.tif]

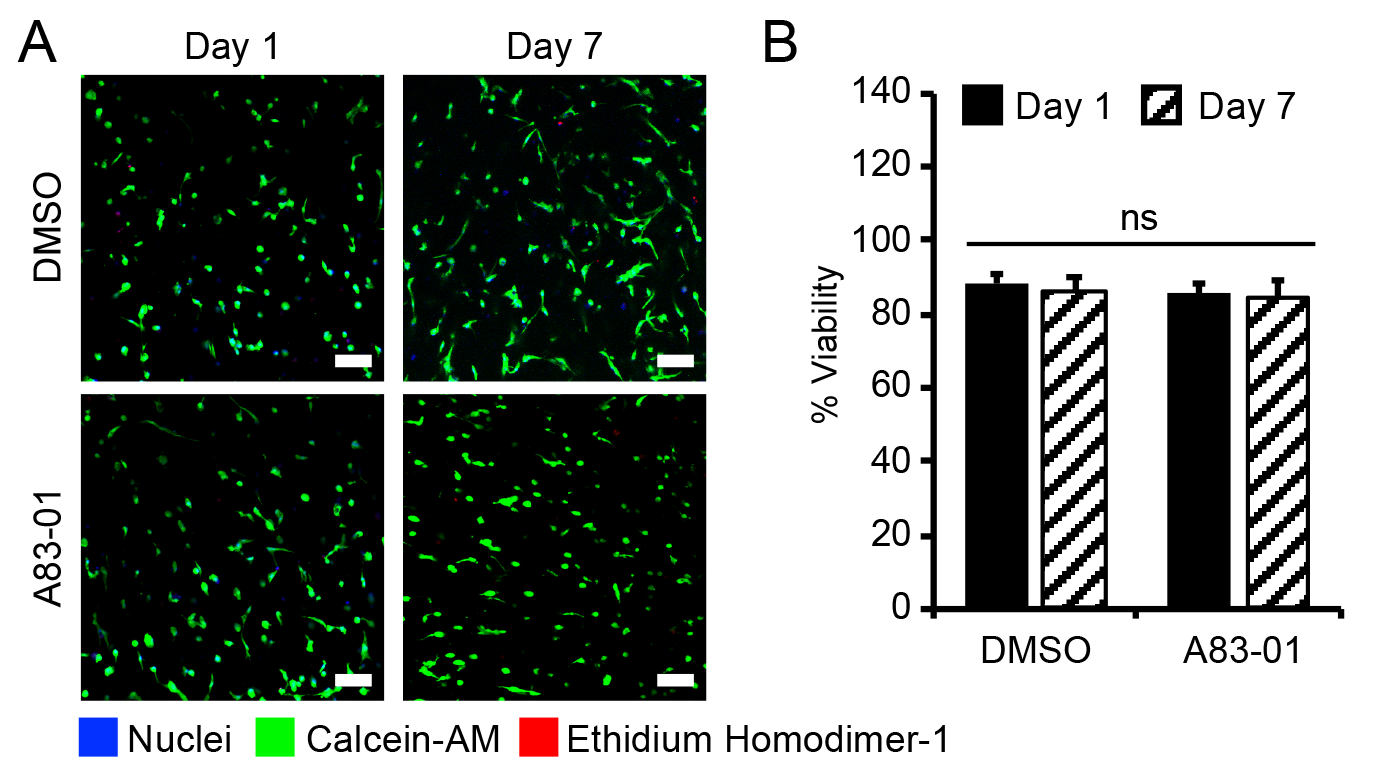

Supplement: S11 Fig — (A) Representative live/dead images of 3:3 MVEC:AoAF co-cultures in 7.5wt% hydrogels with 3 mM RGD after 1 and 7 days of culture with 1 μM A83-01 or DMSO (control). Green indicates live cell bodies and red indicates nuclei in necrotic cells. Scale bar = 100 μm. (B) Over 85% viability was observed for vascular cells encapsulated PEG hydrogels after 1 day of treatment with 1 μM A83-01 or DMSO (control). High viability was maintained (>85%) in 3:3 co-cultures even after 7 days of treatment with 1 μM A83-01 or DMSO (control). In B: data are represented as the mean ± SEM, with n = 3 biological replicates per condition. A repeated measures one-way ANOVA was used to detect statistical significance. (TIF) [file pone.0244243.s011.tif]

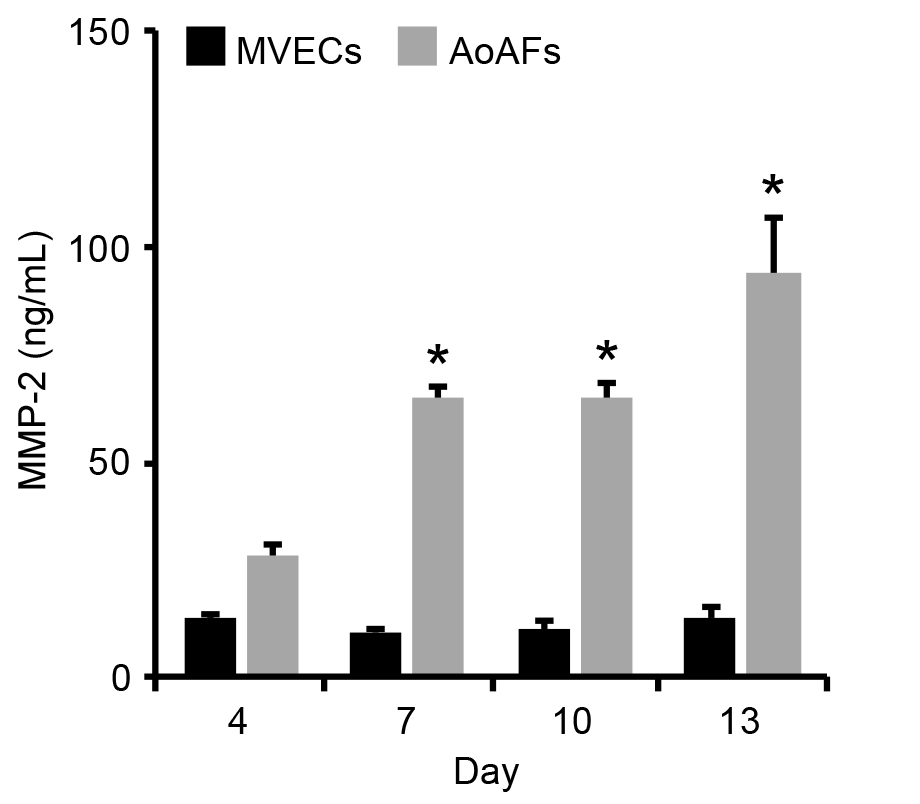

Supplement: S12 Fig — Encapsulated AoAFs produced significantly more MMP‐2, as detected via ELISA, compared encapsulated MVECs. MVECs and AoAFs were encapsulated independently at 3x10^6^ cells/mL of 7.5wt% hydrogel containing 3 mM RGD. Conditioned medium was collected every 3 days and stored at -80°C until analysis. Data are represented as the mean ± SEM, with n = 3 biological replicates per condition. A repeated measures two-way ANOVA, followed by a Tukey HSD post hoc test, was used to detect statistical significance, *p<0.05 for AoAFs relative to MVECs. (TIF) [file pone.0244243.s012.tif]

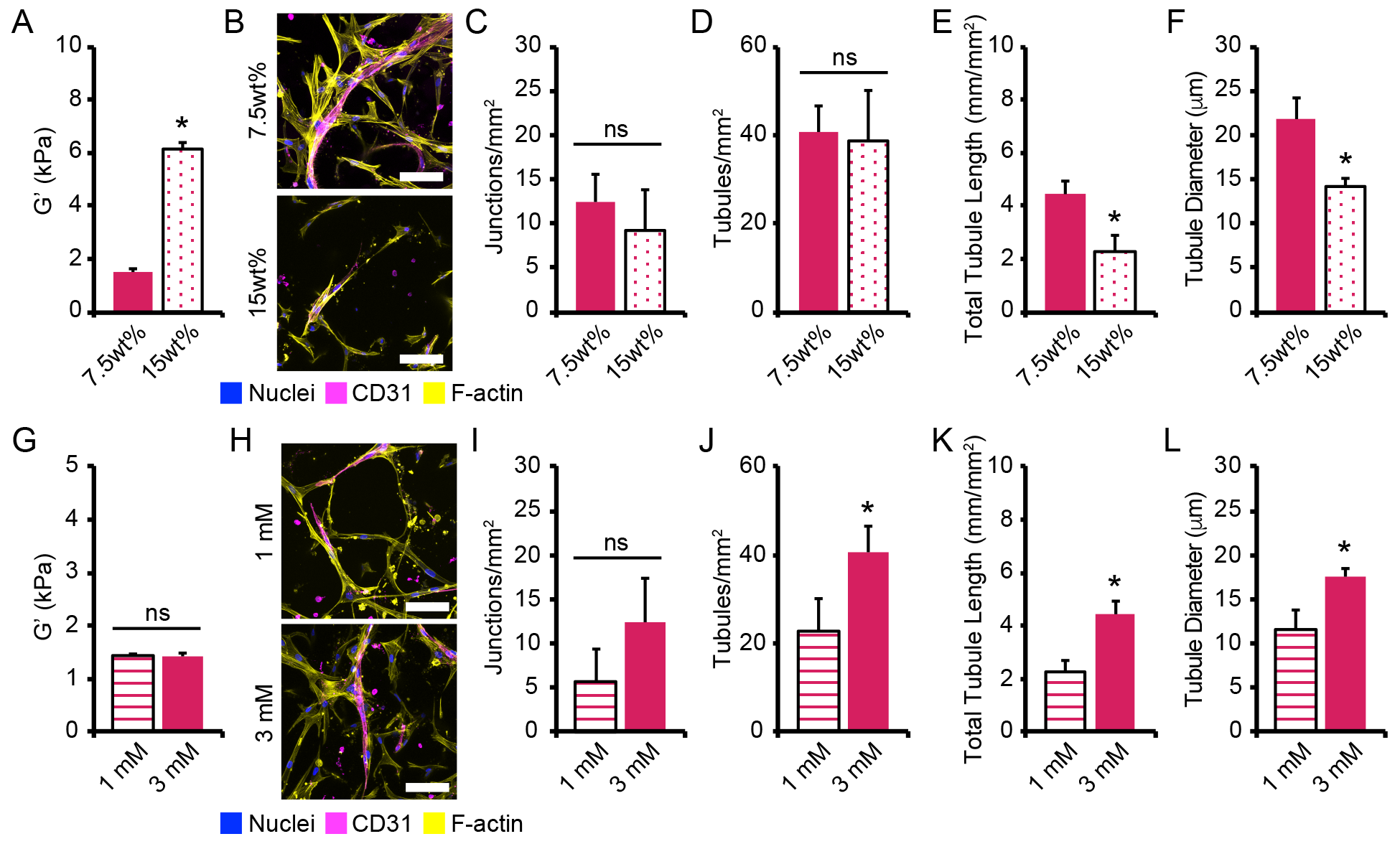

Supplement: S13 Fig — (A) The initial equilibrium storage moduli (G’, kPa), evaluated by oscillatory shear rheology after 24 h, significantly increased with increasing wt%. (B) Representative images of microvascular tubules in 7.5wt% and 15wt% hydrogels after 14 days of culture. MVECs are depicted by CD31 (magenta), while F-actin is stained with phalloidin-568 (yellow) and nuclei are counterstained with Hoechst 33258 (blue). Scale bar = 100 μm. Increasing hydrogel wt% did not impact (C) the number of junctions per mm2 or the (D) the number of MVEC tubules per mm2 after 14 days of culture. (E) Total tubule length (mm/mm2) and (F) tubule diameter decreased significantly as hydrogel wt% increased. (G) The initial equilibrium storage moduli (G’, kPa), evaluated by oscillatory shear rheology after 24 h, was similar for 7.5wt% hydrogels containing either 1 mM or 3 mM RGD. (H) Representative images of microvascular tubules in 7.5wt% hydrogels with 1 or 3 mM RGD after 14 days of culture. MVECs are depicted by CD31 (magenta), while F-actin is stained with phalloidin-568 (yellow) and nuclei are counterstained with Hoechst 33258 (blue). Scale bar = 100 μm. (I) RGD concentration did not impact the number of junctions per mm2 between MVEC tubules. (J) The number of tubules per mm2 and (K) total tubule length (mm/mm2) increased significantly with increasing RGD concentration after 14 days of culture. (L) Tubule diameter on day 14 significantly increased with increasing RGD concentration. In A, C-G, I-L: data are represented as the mean ± SEM, with n = 3 biological replicates per condition. An unpaired student’s t-test detect statistical significance, *p<0.05 for 7.5 wt% hydrogels containing 3 mM RGD relative to either (A, C-F) 15wt% hydrogels containing 3 mM RGD or (G, I-L) 7.5wt% hydrogels containing 1 mM RGD. (TIF) [file pone.0244243.s013.tif]

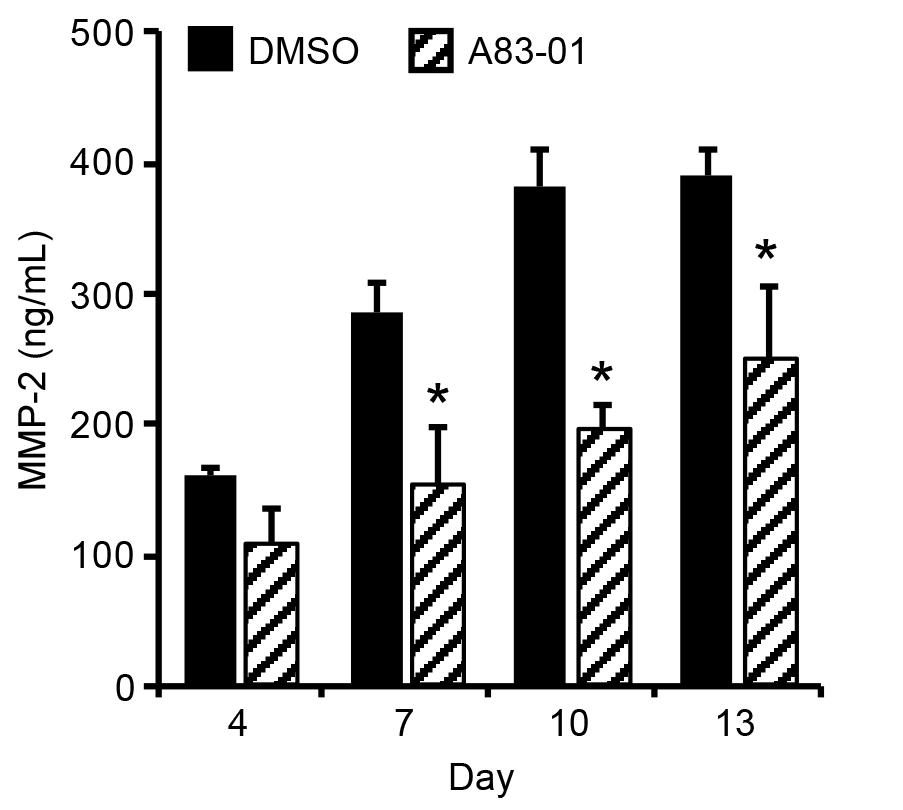

Supplement: S14 Fig — MMP-2 production was decreased in co-cultures treated with 1 μM A83-01 compared to DMSO-treated controls, as detected via ELISA. Data are represented as the mean ± SEM, with n = 3 biological replicates per condition. A repeated measures two-way ANOVA, followed by a Tukey HSD post hoc test, was used to detect statistical significance, *p<0.05 for control cultures relative to cultures treated with 1 μM A83-01, at a given timepoint. (TIF) [file pone.0244243.s014.tif]
