# Supplementary material for: Regulation of neovasculogenesis in co-cultures of aortic adventitial fibroblasts and microvascular endothelial cells by cell-cell interactions and TGF-β/ALK5 signaling
Source: PLoS One. 2020 Dec 28;15(12):e0244243. doi: 10.1371/journal.pone.0244243 (PMC7769260; doi:10.1371/journal.pone.0244243)
Supplement: S1 Table — (DOCX) [file pone.0244243.s015.docx]

**S1 Table.** Antibodies used for immunostaining of MVECs and AoAFs.

| **Target** | **Antibody** | **Details** | **Dilution** | **Supplier** | **Catalog #** |
| --- | --- | --- | --- | --- | --- |
| **CD31** | Primary | Mouse IgG1 monoclonal [C31.7] | 1:100 | Abcam | ab187376 |
|  | Secondary | Alexa Fluor® 647 goat anti-mouse | 1:200 | Life Technologies | A32728 |
| **Collagen IV** | Primary | Rabbit IgG polyclonal | 1:100 | Abcam | ab6586 |
|  | Secondary | Alexa Fluor® 488 goat anti-rabbit | 1:200 | Life Technologies | A11034 |
| **Laminin** | Primary | Rabbit IgG polyclonal | 1:100 | Abcam | ab11575 |
|  | Secondary | Alexa Fluor® 488 goat anti-rabbit | 1:200 | Life Technologies | A11034 |
| **aSMA-FITC** | Primary | Mouse IgG2a monoclonal [1A4] | 1:100 | Sigma Aldrich | F3777 |
| **TGF-β1** | Primary | Rabbit IgG polyclonal | 1:50 | Abcam | ab92486 |
|  | Secondary | Alexa Fluor® 488 goat anti-rabbit | 1:500 | Life Technologies | A11034 |
| **TGFβR−1** | Primary | Rabbit IgG polyclonal | 1:50 | Abcam | ab31013 |
|  | Secondary | Alexa Fluor® 488 goat anti-rabbit | 1:500 | Life Technologies | A11034 |
| **SMAD-2/3** | Primary | Rabbit IgG  monoclonal [D7G7] | 1:100 | Cell Signaling Technologies | 8685S |
|  | Secondary | Alexa Fluor® 488 goat anti-rabbit | 1:500 | Life Technologies | A11034 |
