# Supplementary material for: Regulation of neovasculogenesis in co-cultures of aortic adventitial fibroblasts and microvascular endothelial cells by cell-cell interactions and TGF-β/ALK5 signaling
Source: PLoS One. 2020 Dec 28;15(12):e0244243. doi: 10.1371/journal.pone.0244243 (PMC7769260; doi:10.1371/journal.pone.0244243)
Supplement: S2 Table — (DOCX) [file pone.0244243.s016.docx]

**S2 Table.** Cell adhesion peptides used in laminin-derived peptide hydrogels.

| **Peptide** | **Sequence** | **Concentration in Hydrogel (mM)** | **MW (Da)** | **Binding Partners** |
| --- | --- | --- | --- | --- |
| RGD | *MI*-KGGRGDSPG | 0.4 | 1021.1 | αvβ3 |
| AG10 | *Ml*-KDRSGNRWHSIYITRFG | 0.1 | 2317.6 | α6β1 |
| AG73 | *MI*-KDRSGRKRLQVQLSIRT | 0.5 | 2265.6 | Syndecan |
| YIGSR | *MI*-GGGYIGSR | 2.0 | 930.1 | α4β1, α6β1, α3β1, 67 kDa Lm receptor |
