# Supplementary material for: Regulation of neovasculogenesis in co-cultures of aortic adventitial fibroblasts and microvascular endothelial cells by cell-cell interactions and TGF-β/ALK5 signaling
Source: PLoS One. 2020 Dec 28;15(12):e0244243. doi: 10.1371/journal.pone.0244243 (PMC7769260; doi:10.1371/journal.pone.0244243)
Supplement: S1 File — A description of all supplemental methods. (PDF) [file pone.0244243.s017.pdf]

Supporting Information

Assessment of Co-Culture Medium on Cells in 2D Culture: The impact of co-culture medium on MVEC and AoAF measures of adhesion and proliferation was assessed in initial 2D experiments. MVECs and AoAFs were independently seeded at a density of 1.5×10^4 cells/cm^2 in a 96 well plate (200 μL medium per well). Cells were cultured in specific medium (EGM2-MV for MVECs; FGM for AoAFs) or co-culture medium (1:1 EGM2-MV:FGM). After 2 hrs, medium was removed and the PrestoBlue® assay was performed to establish a baseline of cell adhesion. Briefly, the PrestoBlue® Cell Viability Reagent (Invitrogen, Waltham, MA) was diluted 1:10 in medium, added to cells, and incubated for 2 hrs at 37°C before reading on the plate reader (λ_{ex} 535 nm, λ_{em} 615 nm). Medium was replaced and cells were cultured for 72 hrs at 37°C and 5% CO_2. Measurements were repeated after 72 hrs and the change in signal was used as an indicator of cell proliferative activity.

MVEC Tube Formation Assay on Matrigel: To assess the effects of the laminin-derived peptides on MVEC tube formation, an Angiogenesis μ-Slide (Ibidi, Planegg, Germany) was coated with 10 μL of growth factor-reduced Matrigel (BD Biosciences, San Jose, CA). MVECs (2×10^4 cells per well; 50 μL per well) were seeded onto the solidified Matrigel in EGM2-MV in the presence or absence of 0.2 mM AG10-MI, AG73-MI, or YIGSR-MI and incubated for 4 hrs at 37°C with 5% CO_2. Tube formation was visualized using a Nikon Eclipse Ti-FL microscope (Nikon, Melville, NY) with a 4x Plan Fluor 0.13 N.A. objective and a Nikon Digital Sight DS-Fi1 digital camera (Nikon) controlled by NIS-Elements AR software (version 3.10; Nikon).

MVEC Attachment Assay: Non-degradable hydrogels were formed by crosslinking PEG-SH_4 with four-arm maleimide-functionalized PEG-MI_4. Hydrogels containing 1 mM RGD-MI were prepared at a concentration of 6wt%, crosslinked at a thiol to maleimide stoichiometric ratio of 1:1, and

comprised 3 mM PEG-SH_4, 2 mM PEG-MI_4, and 1 mM RGD-MI. Hydrogels without RGD-MI were prepared at a concentration of 5wt%, crosslinked at a thiol to maleimide stoichiometric ratio of 3:2, and comprised 3 mM PEG-SH_4 and 2 mM PEG-MI_4. To form hydrogels, PEG-SH_4, PEG-MI_4, and RGD-MI were independently dissolved in buffer (10 mM citrate buffer, pH 4.5) and sterilized by passage through a 0.2 μm nonpyrogenic PVDF filter. Maleimide- and thiol-containing solutions were then mixed together via pipetting. Once mixed via gentle pipetting, 50 μL volumes of hydrogel were added to the surface of a 96-well plate, allowed to crosslink for 20 mins at room temperature, and incubated in HBSS for 2 hrs at room temperature. Following removal of HBSS, hydrogels were washed once with EGM2-MV, and MVECs were seeded on the surface of 50 μL of hydrogel in 96-well plates at a density of 5,000 cells per well in 200 μL of medium and incubated at 37°C with 5% CO_2. At specific time points, cells were visualized using an EVOS® FL Auto Imaging System with a 10x UPlanFL 0.3 N.A. objective, controlled by EVOS® FL Auto software (version 1.6; ThermoFisher), to assess cell attachment.

Assessment of ALK5 Signaling by Cells in 2D Culture: In studies where TGF-β superfamily pathways were investigated, MVECs or AoAFs were independently seeded at a density of $1x10^4$ cells/cm^2 in a 48 well plate (200 μL co-culture medium per well) and allowed to adhere for 24 hrs. Medium was removed and the PrestoBlue® assay was performed, as described above, to establish a baseline of cell numbers. Thereafter, cells were immersed in co-culture medium supplemented with A83-01 (0.5 or 5 μM) or DMSO (control) and incubated for an additional 72 hrs at 37°C and 5% CO_2. The PrestoBlue® assay was then repeated and the change in signal was used as an indicator of increased cell numbers over time. In a tandem set of experiments, MVECs or AoAFs were seeded and cultured with A83-01 or DMSO as described above, but not subjected to the PrestoBlue® assay. After culture with A83-01 or DMSO for 72 hrs, cells were fixed with 4% paraformaldehyde in PBS (pH 7.4) in preparation for immunostaining.

Immunostaining of 2D cultures: For assessment of 2D monocultures, MVECs and AoAFs were fixed with 4% paraformaldehyde in PBS (pH 7.4) for 30 mins, permeabilized with 0.1% Triton X-100 for 15 min, and blocked with 3% bovine serum albumin in PBS for 30 min. Antibodies are listed in **S1 Table**. Samples were stained for 1 hr at room temperature with primary antibodies with shaking, washed with PBS, and incubated with secondary antibodies, Phalloidin-568 (1:500), and the nuclear stain Hoechst 33258 (1:1000) for 1 hr at room temperature with shaking. Cells were visualized using an EVOS® FL Auto Imaging System with a 20x LPlanFL 0.4 N.A. objective, controlled by EVOS® FL Auto software (version 1.6; ThermoFisher). Negative controls (e.g. cells not exposed to primary antibodies but incubated with secondaries) were used to validate immunofluorescence signals.

MMP-2 Quantification: Conditioned medium was collected and stored at -80°C until analysis. MMP-2 levels in the medium collected from mono- and co-cultures were quantified via the human MMP-2 Quantikine ELISA kit (R&D Systems, Minneapolis, MN). All assays were used in accordance with the manufacturers' instructions.

Formulation of Alterative Hydrogels: Degradable 7.5wt% hydrogels containing 1 mM RGD-MI were formed by crosslinking PEG-SH_4 (5.8 mM) with PQ-MI_2 (10.1 mM) and RGD-MI (1 mM), while degradable 15wt% hydrogels containing 3 mM RGD-MI, were formed by crosslinking PEG-SH_4 (11.6 mM) with PQ-MI_2 (21.7 mM) and RGD-MI (3 mM). 7.5wt% hydrogels containing 1 mM RGD-MI and 15wt% hydrogels containing 3 mM RGD-MI were crosslinked at a thiol to maleimide stoichiometric ratio of 1.1:1 and 1:1, respectively. All hydrogels were formed by independently dissolving PEG-SH_4, PQ-MI_2, and the cell adhesive peptides in buffer (10 mM sodium phosphate monobasic monohydrate, 5 mM citric acid trisodium salt (anhydrous), 140 mM sodium chloride, pH 4.8) and sterilizing by passage through a 0.2 μm nonpyrogenic PVDF filter. Maleimide- and

thiol-containing solutions, along with 10 mM HEPES in HBSS (1/5 of the final hydrogel volume, pH 7.4), were then mixed together via pipetting. Hydrogels were prepared for mechanical characterization, as described in the main text. After 24 h of incubation at 37°C in co-culture medium, the equilibrium shear storage moduli for acellular 7.5wt% containing 1 mM RGD-MI, and 15wt% hydrogels containing 3 mM RGD-MI were 1.4 $\pm$ 0.1 kPa and 6.1 $\pm$ 0.3 kPa, respectively.

To create cell-laden degradable hydrogels, cells were suspended in 10 mM HEPES in HBSS and mixed via gentle pipetting into the solution of hydrogel precursor polymers prior to crosslinking. For 7.5wt% hydrogels with 1 mM RGD-MI or 15wt% hydrogels with 3 mM RGD-MI, MVECs were encapsulated to achieve $3x10^6$ MVECs/mL hydrogel and AoAFs were encapsulated to achieve $3x10^6$ AoAFs/mL hydrogel. Once mixed via gentle pipetting, 10 μL volumes of hydrogel were added to the surface of an 8 well Nunc™ Lab-Tek™ chambered coverglass and allowed to crosslink for 20 mins at room temperature. Hydrogels were then immersed in co-culture medium (400 μL) and incubated at 37°C with 5% CO_2. Medium was replaced every 3 days over the duration of these experiments.
